# Dynamic Changes to the Skeletal Muscle Proteome and Ubiquitinome Induced by the E3 Ligase, ASB2β

**DOI:** 10.1016/j.mcpro.2021.100050

**Published:** 2021-01-29

**Authors:** Craig A. Goodman, Jonathan R. Davey, Adam Hagg, Benjamin L. Parker, Paul Gregorevic

**Affiliations:** 1 Department of Physiology, Centre for Muscle Research (CMR), The University of Melbourne, Victoria, Australia; 2 Australian Institute for Musculoskeletal Science (AIMSS), Sunshine Hospital, The University of Melbourne, St Albans, Victoria, Australia; 3 Baker Heart and Diabetes Institute, Melbourne, Victoria, Australia; 4 Department of Physiology, Monash Biomedicine Discovery Institute, Monash University, Clayton, Victoria, Australia; 5 Charles Perkins Centre, School of Life and Environmental Science, The University of Sydney, Sydney, NSW, Australia; 6 Department of Biochemistry and Molecular Biology, Monash University, Clayton, Victoria, Australia; 7 Department of Neurology, The University of Washington School of Medicine, Seattle, Washington, USA

**Keywords:** skeletal muscle atrophy, protein degradation, muscle contraction, ubiquitination, proteasome, adeno-associated viral vectors, autophagy, mitochondria, filamin, titin, 2D nano-UHPL-MS/MS, two-dimensional nano-ultrahigh-performance mass spectrometry, CMV, cytomegalovirus, CSA, cross-sectional area, diGly, di-glycine, dSOCS, deleted C-terminal SOCS box domain, DUB, deubiquitinase, E-C, excitation-contraction, EDL, extensor digitorum longus, ETC, electron transport chain, GFP, green fluorescent protein, GO, gene ontology, HBSS, Hank’s buffered saline solution, MCS, multiple cloning site, mTORC1, mechanistic target of rapamycin complex 1, OMM, outer mitochondrial membrane, PVDF, polyvinylidene difluoride, rAAV, recombinant adeno-associated virus, ROS, reactive oxygen species, SOCS, suppressor of cytokine signaling, TA, tibialis anterior, TCA, tricarboxylic acid, TEAB, tetraethylammonium tetrahydroborate, TFA, trifluoroacetic acid, TMT, tandem mass tag, UIM, ubiquitin-interacting motif, UPS, ubiquitin proteasome system, WT, wild type

## Abstract

Ubiquitination is a posttranslational protein modification that has been shown to have a range of effects, including regulation of protein function, interaction, localization, and degradation. We have previously shown that the muscle-specific ubiquitin E3 ligase, ASB2β, is downregulated in models of muscle growth and that overexpression ASB2β is sufficient to induce muscle atrophy. To gain insight into the effects of increased ASB2β expression on skeletal muscle mass and function, we used liquid chromatography coupled to tandem mass spectrometry to investigate ASB2β-mediated changes to the skeletal muscle proteome and ubiquitinome, *via* a parallel analysis of remnant diGly-modified peptides. The results show that viral vector-mediated ASB2β overexpression in murine muscles causes progressive muscle atrophy and impairment of force-producing capacity, while ASB2β knockdown induces mild muscle hypertrophy. ASB2β-induced muscle atrophy and dysfunction were associated with the early downregulation of mitochondrial and contractile protein abundance and the upregulation of proteins involved in proteasome-mediated protein degradation (including other E3 ligases), protein synthesis, and the cytoskeleton/sarcomere. The overexpression ASB2β also resulted in marked changes in protein ubiquitination; however, there was no simple relationship between changes in ubiquitination status and protein abundance. To investigate proteins that interact with ASB2β and, therefore, potential ASB2β targets, Flag-tagged wild-type ASB2β, and a mutant ASB2β lacking the C-terminal SOCS box domain (dSOCS) were immunoprecipitated from C2C12 myotubes and subjected to label-free proteomic analysis to determine the ASB2β interactome. ASB2β was found to interact with a range of cytoskeletal and nuclear proteins. When combined with the *in vivo* ubiquitinomic data, our studies have identified novel putative ASB2β target substrates that warrant further investigation. These findings provide novel insight into the complexity of proteome and ubiquitinome changes that occur during E3 ligase-mediated skeletal muscle atrophy and dysfunction.

## Highlights

• Proteomic and ubiquitinomic analysis of increased ASB2β in skeletal muscle
• ASB2β increased the abundance of proteome and cytoskeletal proteins
• ASB2β reduced the abundance of mitochondrial and contractile proteins
• No simple relationship between changes in protein abundance and ubiquitination

## Graphical abstract

**Figure:**
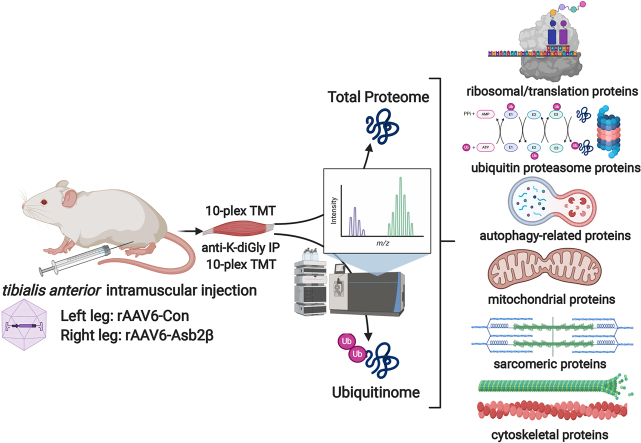

## In Brief

The E3 ubiquitin ligase ASB2β has been identified as a regulator of skeletal muscle mass. To gain insights into potential mechanisms of action, mouse muscles expressing a Flag-tagged ASB2β were investigated using quantitative proteomic methods. The results identified ASB2β-induced changes in the abundance and ubiquitination of proteins associated with mitochondria, the sarcomere, and the cytoskeleton. Additional *in vitro* studies identified novel putative ASB2β target substrates. The results highlight the complex relationship between protein abundance and ubiquitination in ASB2β-mediated muscle adaptation.

Skeletal muscle mass is broadly regulated by the net difference between the global rates of protein synthesis and protein degradation, with a net increase in protein degradation eventually leading to reduced muscle mass (*i.e.*, muscle atrophy/wasting) (1). Cellular proteins can be degraded *via* several mechanisms, including by calcium-activated proteases (*i.e.*, calpains), cysteine-aspartic proteases (*i.e.*, caspases), or through autophagy; however, the majority of proteins are degraded by the ubiquitin proteasome system (UPS) (2, 3, 4).

Typically, for a protein to be targeted for degradation by the proteasome, it is first covalently modified at specific lysine residues by the addition of a linear chain of ubiquitin proteins (polyubiquitination), with each successive ubiquitin covalently linked to the previous ubiquitin at lysine 48 (K48) (5). However, not all ubiquitination events lead to protein degradation. For example, proteins can also be monoubiquitinated (at single or multiple sites) or polyubiquitinated with linear or branched chains linked *via* several different ubiquitin lysine residues (*e.g.*, K6, K11, K27, K29, K33, K63), leading to changes in protein function, localization, protein-to-protein interactions, and signaling [For reviews see (6, 7, 8)]. Ubiquitination is an ATP-dependent process that requires the sequential action of three classes of proteins known as E1 ubiquitin-activating enzymes, E2 ubiquitin-conjugating enzymes, and E3 ubiquitin protein ligases (9). An active E3 ligase is a multiprotein complex that includes a scaffold protein that allows the binding of other key proteins that ultimately facilitate the docking of specific target proteins and the E2 enzyme. Once the target protein is bound to the E3 complex, ubiquitin is transferred to the target either directly by the E2 enzyme or from the E2 to the E3 and then to the target (10). Impressively, there are ∼600 E3 ligases encoded in the human genome (10). Furthermore, there are at least 99 deubiquitinase (DUB) enzymes (11). Together, these candidates suggest a high degree of complexity in the regulation of the ubiquitiome and the requirement for processes that control target specificity, including *via* spatial and temporal regulation of expression and activation.

In skeletal muscle, the expression of specific E3 ligases has been shown to be upregulated in atrophic conditions, including the muscle-specific E3 ligases, MuRF1 (Trim63) and atrogin-1 (Fbxo32 or MAFbx) (12, 13, 14), and more recently, MUSA1 [Fbxo30 (15)]. Importantly, the knockdown/knockout of these E3 ligases renders varying degrees of resistance to muscle atrophy, suggesting that they play a role in the atrophic response (12, 15). Another muscle-specific E3 ligase recently implicated in the regulation of muscle mass is the β isoform of the ankyrin repeat-containing protein with a suppressor of cytokine signaling box-2 (ASB2β) (16). As the name suggests, ASB2 proteins (α and β isoforms) are made up of a series of ankyrin motifs and a C-terminal suppressor of cytokine signaling (SOCS) box domain, which, in turn, contains an Elongin B/C (BC) box and a Cullin5 (Cul5) box. The distinguishing feature between the α and β isoforms of ASB2 is the presence of extra 48 amino acids at the N terminus of ASB2β that contains a ubiquitin-interacting motif (UIM), which may play a role in ASB2β ubiquitination and in binding to other polyubiquitinated proteins (16, 17, 18). For the formation of an active ASB2 E3 ligase complex, the scaffold protein, Cul5, binds to ASB2’s Cul5 box, and an Elongin B/C heterodimer binds to the BC box, stabilizing the ASB2/Cul5 interaction (19). ASB2’s ankyrin repeats play a role in target protein recruitment, while Cul5 recruits the RING finger protein, Rbx2, which in turn allows the recruitment of the E2 enzyme (20). Recent studies in cardiac muscle cells have shown that ASB2β is localized to the sarcomeric Z disc and stimulates the degradation of sarcomere-associated proteins, desmin and filamin A, suggesting that ASB2β might play a role in regulating sarcomere/cytoskeletal structure and function (21, 22).

In regard to ASB2β’s function in skeletal muscle, an increase in its expression has been shown to be essential for myoblast differentiation *in vitro* due to its ability to ubiquitinate and target for degradation, another sarcomere-interacting cytoskeletal protein, filamin B (16). More recently, we have shown that ASB2β mRNA and protein are markedly decreased in a mouse model of muscle hypertrophy induced by follistatin (FST) overexpression (23). We observed that ASB2β was suppressed approximately twofold after only 2 days of induced FST expression, suggesting that a reduction in ASB2β might be required for muscle hypertrophy (23). Consistent with this hypothesis, another recent study showed that ASB2β expression was reduced in a mouse model of cardiac hypertrophy (21). ASB2β expression in skeletal muscle appears to be regulated, in part, by a Smad2/3 transcription factor-dependent mechanism, with the overexpression of the Smad2/3 inhibitor, Smad7, or combined overexpression of activin B and myostatin prodomains (inhibitors of activin/myostatin-induced Smad2/3 signaling), leading to marked suppression of ASB2β mRNA (23, 24). Given that Smad2/3 signaling is able to stimulate muscle atrophy (25, 26, 27, 28), these data suggest that ASB2β may play a role in Smad2/3-mediated muscle atrophy. Consistent with the idea that ASB2β may play a role in muscle atrophy, we recently found that the overexpression of ASB2β in mouse muscle was sufficient to induce a decrease in muscle mass (23). In this context, it is interesting to note that ASB2β was recently shown to be upregulated in proliferating myoblasts and differentiated myotubes obtained from patients with type 1 myotonic dystrophy (DM1), a neuromuscular disease whose features include muscle atrophy and weakness (29, 30). When considered together, these findings provided a compelling rationale for examining how increased expression of ASB2β might alter the skeletal muscle proteome and ubiquitinome.

Therefore, to gain insight into how increased ASB2β expression might affect skeletal muscle mass and function, we utilized two-dimensional nano-ultrahigh-performance mass spectrometry (2D nano-UHPL-MS/MS)-based quantitative proteomics to investigate ASB2β-induced changes to the mammalian muscle proteome and whether these were associated with changes in protein ubiquitination. Using immunoprecipitation of wild type (WT) and mutant forms of ASB2β from C2C12 myotubes, combined with label-free proteomics, we also identified ASB2β interacting proteins and novel putative ASB2β target substrates.

## Experimental Procedures

### Generation of rAAV6 Vectors

Recombinant adeno-associated viral vectors (AAVs) expressing ASB2β constructs were generated as described previously (31). In brief, rAAV6 plasmids containing cDNA constructs were cotransfected with pDGM6 packaging plasmid into HEK293 cells (seeded 16 h prior) using the calcium phosphate precipitate method to generate type-6 pseudotyped viral vectors. After 72 h, cells and culture medium were collected and subjected to three freeze–thaw cycles, before clarification, using a 0.22 mm filter (EMD Millipore). Vectors were purified by affinity chromatography using a heparin column (HiTrap, GE Healthcare) and concentrated by ultracentrifugation overnight, with resuspension in sterile Ringer’s solution. Vector concentration was determined using a customized qPCR reaction (Applied Biosystems).

### Animal Experiments

*In vivo* procedures were conducted in accordance with the relevant codes of practice for the care and use of animals for scientific purposes (National Institute of Health, 1985, and the National Health & Medical Research Council of Australia, 2013). All experimental protocols were approved by the Alfred Medical and Education Precinct Animal Ethics Committee and the University of Melbourne Animal Ethics Committee. All surgical procedures were performed under inhalation of isoflurane in medical oxygen with postoperative analgesia. Cohorts of 8 to 10 week-old male and female C57Bl/6 mice were used for all experiments. Mice were fed standard chow diets with access to drinking water *ad libitum* while housed under a 12-h light–dark cycle. For experiments related to the effect of ASB2β overexpression on muscle mass, muscle fiber cross-sectional area and type, muscle function, and western blot analysis, doses of recombinant AAV6 vectors containing expression cassettes for WT flag-tagged ASB2β [rAAV: ASB2β; 5 × 10^9^ vector genomes (vg), (23)] were diluted in 30 μl of Hank’s buffered saline solution (HBSS) and directly injected into the anterior compartment of the mouse lower hindlimb, which enables transduction of the tibialis anterior (TA) and extensor digitorum longus (EDL) muscles. Control injections consisted of the administration of a viral vector lacking a functional gene [rAAV:Control (CON); 5 × 10^9^ vg] into the contralateral limb. For the proteomic/ubiquitinomic experiments, hindlimbs were injected with a 1 × 10^9^ dose of rAAV:ASB2β vg, or rAAV:CON in the contralateral control limb, and the TA muscles were collected 10 days postinjection. For ASB2β knockdown experiments, two in series ASB2β shRNAs (5’-TGAACAGGTTGCTGCTATACTGTTTTGGCCACTGACTGACAGTATAGCCAACCTGTTCA-3’ and 5’-AATGCTGTCTCTTCCTGCAGGTTTTGGCCACTGACTGACCTGCAGGAAGACAGCATTA-3’) were synthesized within the sequence encoding miR-155 and subcloned into a pAAV6:CMV-humanized Renilla GFP-SV40pA plasmid. rAAV:ASB2β shRNA or control rAAV:LacZ shRNA (5 × 10^9^ vg) was diluted in 30 μl of HBSS and directly injected into the anterior compartment muscles of the hindlimb. TA or EDL muscles were collected at 7, 14, 28, or 84 days post-rAAV injection, with mice being humanely killed *via* cervical dislocation and the muscles rapidly excised and weighed before subsequent processing.

### Protein Extraction and Western Blotting

Muscles were homogenized in NP-40 lysis buffer containing protease and phosphatase inhibitor cocktails. Lysates were centrifuged at 15,000*g* for 20 min at 4 °C, protein concentration was determined using a BCA protein assay kit (Thermo Scientific) and samples denatured for 5 min at 95 °C. Protein fractions were resolved by SDS-PAGE using pre-cast 4 to 12% Bis-Tris gels (Life Technologies), blotted onto polyvinylidene difluoride membranes (Millipore). Membranes were incubated with primary antibodies (anti-Flag (Sigma, F1804, 1:1000), anti-ASB2 (Sigma, SAB2701121, 1:1000), anti-p70^S6k1^ (CST, 9202, 1:1000), anti-phospho-p70^S6k1^ T389 (CST, 9205, 1:1000), anti-LC3B (CST, 2775, 1:1000), anti-AMPKα (CST, 2532, 1:1000), and anti-phospho-AMPKα T172 (CST, 2535, 1:1000)). Bound primary antibodies were detected by incubation with an HRP-conjugated secondary antibody (Bio-Rad) in 5% skim milk powder. Chemiluminescence was detected using ECL western blotting detection reagents (GE Healthcare, Buckinghamshire, United Kingdom) and Immobilon Forte Western HRP substrate (Merck, New Jersey, United States). Quantification of western blots was performed using ImageJ software (http://rsb.info.nih.gov/ij/index.html) or Fusion CAPT Advance software (Vilber Lourmat). All samples were run on the same membrane. Membranes were stripped of phospho-antibodies and subsequently reprobed for total protein. All quantified phospho-proteins were expressed as a ratio with total protein from the same membrane (*i.e.*, phospho:total ratio for p70^S6k1^, AMPK and Drp1), while the lipidated form of LC3B (LC3BII) was expressed as a ratio with LC3BI from the same membrane. All other proteins were normalized to the signal for total protein obtained from Stain-Free gels (Bio-Rad) or Ponceau staining.

### Immunofluorescent Analysis of Muscle Fiber CSA and Muscle Fiber Type

The analysis of myofiber cross-sectional area (CSA) was carried out on 8 μm TA or EDL muscle cryosections fixed in 4% PFA for 10 min prior to blocking with goat serum (5% goat serum, 2% BSA and 0.1% Triton-X in PBS) for 1 h at room temperature. Blocked sections were incubated with the following primary antibodies in PBS containing 0.05% Tween-20 (PBST) overnight. TA muscle sections were incubated with anti-laminin (rabbit IgG, 1:25, Sigma, L9393) for measurements of fiber CSA-independent of muscle fiber type. EDL sections were incubated with anti-laminin, anti-myosin heavy chain (MHC) type 2a (mouse IgG1, 1:25, DSHB, SC-71), and anti-MHC type 2B (mouse IgM, 1:10, DSHB, BF-F3) (Note—as mouse EDL muscles are composed of type 2A, 2D/X, and 2B fibers (32), any EDL fibers that did not label for type 2a and 2b MHC were deduced to be MHC 2 days/x expressing fibers.) Labeled sections were washed in PBST and incubated with the following secondary antibodies in PBST: Alexa Fluor 555 anti-mouse IgG1 (1:250), Alexa Fluor 350 anti-mouse IgM (1:25), and Alexa Fluor 647 anti-rabbit IgG (1:250). Fully labeled sections were coverslipped in Mowiol 4-88 mountant and imaged using a fluorescence microscope (Axio Imager M2, Zeiss). Muscle fiber CSA was determined using ImageJ software (US National Institutes of Health, Bethesda, MD, USA) by measuring at least 400 fibers per muscle.

### *In Vivo* Assessment of Skeletal Muscle Force-Producing Capacity

Torque production capacity of the anterior compartment of the hindlimb was analyzed *in vivo* using a dual-mode force transducer (300C, Aurora Scientific) with a footplate attachment. Mice were anesthetized with tribromoethanol (200–300 mg/kg), and the hind limbs were shaved prior to placement in a supine position on a thermostatically controlled stage. The knee was clamped in a stationary position, and the foot was firmly fixed to the footplate using adhesive tape. Monopolar needle electrodes were placed subcutaneously at the proximal end of the anterior compartment of the hind limb to flank the tibial nerve. Torque–frequency curves were obtained by incrementally increasing stimulation frequencies, with a pulse width of 0.2 ms and a duration of 0.2 s, pausing for 60 s between stimulations. Stimulations were controlled and analyzed using purpose-built software (DMCv5.5 and DMAv5.3, respectively, Aurora Scientific).

### Muscle Peptide Preparation, Isobaric Labeling, and Immunoprecipitation

Frozen muscle samples were homogenized in 6 M urea, 2 M thiourea, 0.1% SDS, 25 mM triethylammonium bicarbonate (TEAB) pH 7.9 containing 20 mM sodium pyrophosphate, 10 mM sodium fluoride, 2 mM sodium orthovanadate, and a protease inhibitor cocktail (11873580001, Roche) by tip-probe sonication and centrifuged at 16,000*g* for 15 min at 4 °C. Lysates were precipitated with five volumes of acetone overnight at −30 °C. Protein pellets were centrifuged at 10,000*g*, 10 min at 4 °C resuspended in 6 M urea, 2 M thiourea, 25 mM TEAB, pH 7.9 and quantified by fluorescence (Qubit Q33212, Thermo Fisher). Concentrations were normalized and 600 μg of protein reduced with 10 mM dithiothreitol (DTT) for 60 min at 25 °C followed by alkylation with 40 mM chloroacetamide for 30 min at 25 °C in the dark. The reaction was quenched to a final concentration of 20 mM dithiothreitol and digested with lysyl endopeptidase Lys-C (125-05061, Wako Pure Chemical Industries) at 1:50 enzyme to substrate ratio for 2 h at 25 °C. The mixture was diluted fivefold with 25 mM TEAB and digested with trypsin (V5111, Promega; trypsin was selected with protease specificity C terminal to Lys and Arg except proceeded by a proline) at 1:50 enzyme-to-substrate ratio for 16 h at 30 °C. The peptide mixture was acidified to a final concentration of 2% formic acid and centrifuged at 16,000*g* for 15 min. Peptides were desalted using hydrophilic–lipophilic balance-solid-phase extraction (HLB-SPE) (186000128, Waters) and eluted with 50% acetonitrile, 0.1% TFA. The purified peptides were aliquoted (95% for diGly immunoprecipitation analysis and 5% for total proteome analysis) and dried by vacuum centrifugation. Immunoprecipitation of diGly-containing peptides was performed as described previously (33, 34, 35) by resuspending the peptides in IP buffer (50 mM MOPs, 10 mM Na2HPO4, 50 mM NaCl2, pH 7.2) and incubated with agarose-conjugated anti-diGly antibody [CST, 5562, PTMScan Ubiquitin Remnant Motif (K-ε-GG)] overnight at 4 °C with rotation. The beads were washed four times with IP buffer followed by water and eluted with 0.2% TFA and dried by vacuum centrifugation. Immunoprecipitated peptides and peptides for total proteome analysis were resuspended in 100 mM HEPES, pH 7.6, and labeled with 10-plex Tandem Mass Tags (TMT) for 1.5 h at room temperature followed by deacylation with 0.25% hydroxylamine for 15 min and quenching with 0.1% TFA. Peptides from each 10-plex experiment were pooled and desalted using HLB-SPE and dried by vacuum centrifugation. Peptides were fractionated (12 fractions for total proteome analysis and six fractions for diGly-peptide analysis) on an in-house packed TSKgel amide-80 HILIC column as previously described (36).

### *In Vivo* Proteomic and Ubiquitinomic Mass Spectrometry and Data Analysis

Peptides were resuspended in 2% acetonitrile, 0.1% formic acid, and loaded onto a 50 cm × 75 μm inner diameter column packed in-house with 1.9 μm C18AQ particles (Dr Maisch GmbH HPLC) using Dionex nano-UHPLC. Peptides were separated using a linear gradient of 5 to 30% Buffer B over 120 min at 300 nl/min (Buffer A = 0.1% formic acid; Buffer B = 80% acetonitrile, 0.1% formic acid). The column was maintained at 50 °C using a PRSO-V1 ion-source (Sonation) coupled directly to a mass spectrometer (Q-Exactive Plus MS). A full-scan MS1 was measured at 70,000 resolution at 200 *m*/*z* (350–1550 *m*/*z*; 100 ms injection time; 3e6 AGC target) followed by isolation of up to 20 most abundant precursor ions for MS/MS (1.2 *m*/*z* isolation; 32 normalized collision energy; 35,000 resolution; 120 ms injection time; 2e5 AGC target). Mass spectrometry data were processed using Proteome Discoverer v2.1 and searched with Sequest HT against the mouse UniProt database (May 2016; 58,239 entries). The data were searched with a maximum of three miscleavages and methionine oxidation, lysine TMT and lysine diGly-TMT set to variable modifications. Carbamidomethylation of cysteine and peptide N-terminus TMT was set as a fixed modification. The precursor ion mass tolerance was set to 20 ppm and product-ion mass tolerance set to 0.02 Da. All results were filtered to 1% peptide spectral matches false discovery rate (FDR) using Percolator (37) and total proteomic data filtered to 1% FDR using Protein Validator in Proteome Discoverer. The localization of modification sites was performed with PTM-RS (38). All data were normalized to the median of each sample, and diGly peptides were further normalized to total proteome data to investigate changes in ubiquitylation at each site. The diGly-modified peptides and proteins were expressed relative to control (AAV:Con) treated muscles, and *t*-tests were performed in Perseus with Benjamini–Hochberg FDR set to 5% (39). The mass spectrometry proteomics data have been deposited to the ProteomeXchange Consortium *via* the PRIDE partner repository with the data set identifier PXD020040 (Username: reviewer19238@ebi.ac.uk and Password: SmH29Vvk) and includes a list of sample identifiers with the TMT labeling channels (40).

### C2C12 Immunoprecipitation, Mass Spectrometry, and Data Analysis

C2C12 myoblasts were seeded on 6-well plates in growth media (DMEM containing 25 mM glucose, 10% FBS, 4 mM Glutamine) to achieve ∼50% confluence 24 h after seeding. At 24 h postseeding, DNA plasmids encoding Flag-tagged ASB2β (WT Flag-ASB2β) or Flag-tagged ASB2β lacking the C-terminal SOCS box domain (residues 584–634 deleted; dSOCS Flag-ASB2β) were transfected into the myoblasts using transfection reagent following manufacturer’s protocol (TRANS-IT, MirusBio). At 24 h after transfection, the media was changed to differentiation media (DMEM containing 25 mm glucose, 2% horse serum, 4 mM Glutamine), and cells were differentiated over 4 days with replenishment every 48 h. After 4 days of differentiation, myotubes were lysed in 0.3% CHAPS, 150 mM NaCl, 5% glycerol in 50 mM Tris (pH 7.5) containing protease inhibitor cocktail (11873580001, Roche) by passing through a 22- and 27-gauge needle at 4 °C. Cellular debris was removed by centrifugation at 20,000*g*, 10 min at 4 °C and quantified by BCA (23225, Thermo Fisher). Two milligrams of protein was incubated with 40 μl of μMACS Anti-DYKDDDDK beads (130-101-591, Miltenyi Biotech) for 45 min with rotation at 4 °C. The suspension was separated with a μMACS column and magnetic separator and washed with lysis buffer containing only 0.01% CHAPS followed by lysis buffer containing no CHAPS. Proteins were eluted with 2 M urea in 50 mM Tris (pH 7.5) containing 1 mM DTT, 5 mM IAA, and 125 ng of trypsin (V5111 Promega; trypsin was selected with protease specificity C terminal to Lys and Arg except proceeded by a proline) and digested overnight at room temperature. Peptides were acidified to 1% TFA and desalted using Styrene Divinylbenzene–Reversed-Phase Sulfonate (SDB-RPS) microcolumns and eluted with 80% acetonitrile in 2% ammonium hydroxide followed by vacuum concentration. Peptides were resuspended in 2% acetonitrile, 0.1% formic acid, and loaded onto a 50 cm × 75 μm inner diameter column packed in-house with 1.9 μm C18AQ particles (Dr Maisch GmbH HPLC) using Dionex nano-UHPLC. Peptides were separated using a linear gradient of 5 to 30% Buffer B over 90 min at 300 nl/min (Buffer A = 0.1% formic acid; Buffer B = 80% acetonitrile, 0.1% formic acid). The column was maintained at 50 °C using a PRSO-V1 ion-source (Sonation) coupled directly to a Q-Exactive Plus mass spectrometer (MS). A full-scan MS1 was measured at 70,000 resolution at 200 m/z (300–1550 m/z; 100 ms injection time; 3e6 AGC target) followed by isolation of up to 15 most abundant precursor ions for MS/MS (1.2 m/z isolation; 27 normalized collision energy; 17,500 resolution; 55 ms injection time; 1e5 AGC target). A sweep gas was applied during sample loading and reconditioning to prevent contaminant ions entering the mass spectrometer (41). Mass spectrometry data were processed using MaxQuant v1.6.7.0 with all default parameters and searched against the mouse UniProt database (March 2018; 61,665 entries). The data were searched with a maximum of two miscleavages and methionine oxidation and protein N-terminal acetylation set as variable modifications. Carbamidomethylation of cysteine was set as a fixed modification. The precursor ion mass tolerance was set to 20 ppm and product-ion mass tolerance set to 0.02 Da. The MaxLFQ and match-between-runs options were enabled (42). All results were filtered to 1% peptide spectral matches and protein FDR. Proteins were expressed relative to control treated cells, and *t*-tests were performed in Perseus (39) with Benjamini–Hochberg FDR set to 5%. The mass spectrometry proteomics data have been deposited to the ProteomeXchange Consortium *via* the PRIDE partner repository with the data set identifier PXD023391 (Username: reviewer_pxd023391@ebi.ac.uk and Password: jI0yBTIt) and include a list of sample identifiers (40).

### Experimental Design and Statistical Rationale

For statistical analysis not related to the proteomic and ubiquitinomics data sets, data are presented as mean ± SEM, and biological replicates are stated in figure legends. Two groups comparisons (*i.e.*, rAAV:CON *versus* rAAV:ASB2β or control rAAV:LacZ shRNA *versus* rAAV:ASB2β shRNA) were made using a two-way ratio paired *t*-test. Contractile torque–frequency data were analyzed using two-way ANOVA tests to assess statistical differences across multiple conditions, with the Bonferroni post hoc test used for comparisons between the specific group means. Total proteomic and diGly immunoprecipitation was performed from five separate biological replicate mice where the left leg was injected with AAV’s carrying a geneless construct (CON) as a control, and the right leg was injected with AAV’s containing ASB2β. Immunoprecipitation was performed from five or six separate biological replicate cell culture plates. Transfection of C2C12 was performed with the control construct (CON) (n = 5) or the equivalent plasmid carrying the WT FLAG-tagged ASB2β (n = 6) or the equivalent plasmid carrying the dSOCS FLAG-tagged ASB2β construct (n = 5). All differences reported are *p* < 0.05. Statistical analysis was performed using GraphPad Prism 8.4.2.

## Results

### ASB2β Promotes Muscle Atrophy and Impairs Contractile Function

**Fig. 1:**
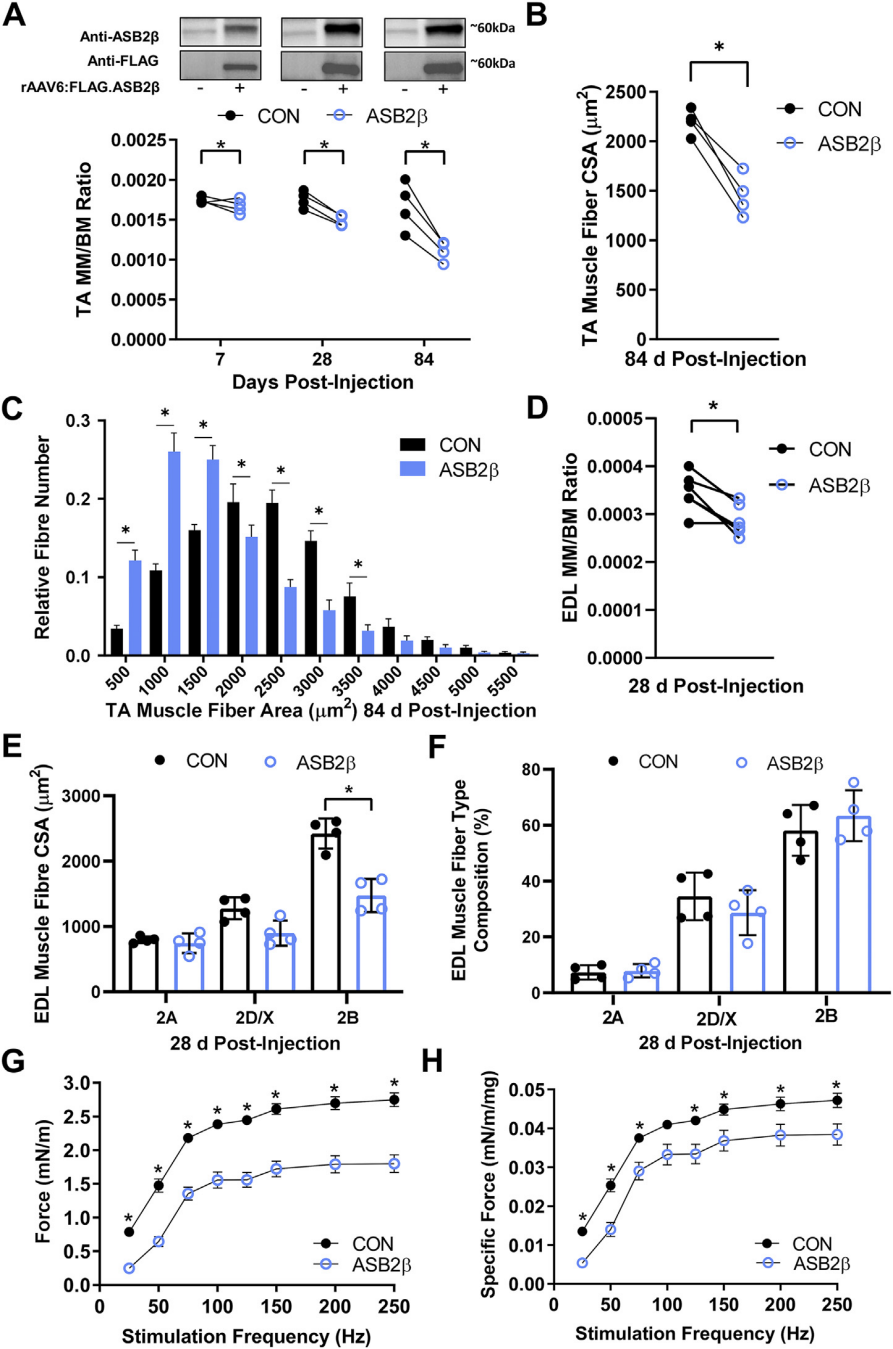
**The effect of ASB2β overexpression on muscle mass, muscle fiber cross-sectional area, muscle fiber type, and *in vivo* force production.** rAAV vectors encoding Flag-tagged ASB2β or a noncoding control (CON) were injected into the anterior compartment of the lower leg of 8 to 10 week-old male C57Bl/6 mice. Tibialis anterior (TA) or extensor digitorum longus (EDL) muscles were dissected at 7, 28 or 84 days postinjection. *A*, the change in TA muscle mass (MM) to body mass (BM) ratio with ASB2β overexpression for 7, 28, and 84 days (n = 4/group). Western blots show Flag-tag and ASB2β expression. *B*, mean TA muscle fiber cross-sectional area (CSA) with 84 days of ASB2β overexpression (n = 4/group). *C*, the relative distribution of TA muscle fiber CSA measurements with 84 days of ASB2β overexpression (n = 4/group). *D*, the change in EDL muscle mass (MM) to body mass (BM) ratio 28 days of ASB2β overexpression (n = 4/group). *E*, mean cross-sectional area (CSA) of EDL fast-twitch type 2A, 2D/X and 2B muscle fibers following 28 days of ASB2β overexpression. *F*, the relative fast-twitch muscle fiber composition of EDL muscles following 28 days of ASB2β overexpression. *G* and *H*, electrically stimulated *in vivo* TA and EDL ankle dorsi-flexion absolute (*G*) and specific (normalized to muscle mass; *H*) torque production following 28 days of ASB2β overexpression (n = 6/group). ∗*p* < 0.05.

Previously, we reported that an intramuscular injection of rAAV6 vectors containing an expression construct for Flag-tagged ASB2β (rAAV:ASB2β) into mouse TA muscles led to a reduction in muscle mass at 14 (∼7%) and 28 days (∼15%) postinjection, when compared with the contralateral muscle injected with rAAV6 vectors containing a geneless control construct (rAAV:CON) (23). As shown in Figure 1*A*, we were able to reproduce our previously reported ∼15% decrease in TA muscle mass in ASB2β expressing muscles at 28 days postinjection (23). To examine the time course of ASB2β-mediated effects, we extended these studies to include earlier and later timepoints. ASB2β-mediated loss of muscle mass commenced rapidly, with a small (∼4%), but significant, decrease in muscle mass observable by 7 days postinjection (Fig. 1*A*). Continued ASB2β expression was associated with a continued decline in muscle mass beyond 28 days, such that by 84 days (12 weeks), ASB2β-expressing TA muscles exhibited a ∼33% reduced mass compared with the contralateral muscles administered rAAV:CON (Fig. 1*A*). We subsequently investigated whether there was a sex-based difference in the ability of ASB2β to promote muscle atrophy and found no difference in the proportion of muscle mass lost at 7, 28, or 84 days postinjection between male and female mice (supplemental Fig. S1). To determine whether the loss of muscle mass was associated with reduced muscle fiber size, we examined muscle fiber CSA in male TA muscles at 84 days postinjection. In close agreement with the muscle mass data, 84 days of ASB2β overexpression resulted in a ∼33% reduction in mean muscle fiber CSA (Fig. 1*B*) and an increased proportion of smaller diameter muscle fibers (Fig. 1*C*). To compare the effects of ASB2β on different muscles, we examined the adjacent EDL muscle at 28 days postinjection and observed a ∼17% decrease in EDL muscle mass (Fig. 1*D*) that was similar to that observed in the TA muscle at the same time point. Interestingly, the loss of EDL muscle mass appeared to be predominantly due to a reduction in the CSA of fast-twitch type 2B muscle fibers (Fig. 1*E*), which occurred in the absence of a change in overall EDL muscle fiber type composition (Fig. 1*F*). Given the ability of ASB2β to promote muscle atrophy, we sought to determine whether reducing ASB2β expression might lead to an increase in muscle mass. In support of this hypothesis, the intramuscular injection of rAAV vectors containing expression constructs encoding for two shRNA sequences that target ASB2β mRNA resulted in the successful knockdown of ASB2β protein 28 days postinjection and a small, but significant, increase in muscle mass at 14 (∼5%) and 28 days (∼7%) postinjection (supplemental Fig. S2). Having determined that ASB2β is a negative regulator of muscle mass, we examined the effect of increased ASB2β expression on muscle function by stimulating TA muscle contractions *in vivo* with at progressively higher stimulation frequencies and measuring the resulting dorsiflexion ankle torque. At 28 days postinjection, muscles administered rAAV:ASB2β produced lower absolute forces at all stimulation frequencies (Fig. 1*G*). When corrected for muscle mass, the muscles overexpressing ASB2β were also observed to generate significantly reduced specific force (Fig. 1*H*). These data show that increased expression of ASB2β is sufficient to induce progressive muscle wasting due to muscle fiber atrophy independent of changes in muscle fiber composition and impair the intrinsic force producing capability of the muscle, independent of muscle size, suggesting the possibility of altered expression and/or function of proteins involved in maintaining muscle fiber structure and contractility.

### Quantitative Analysis of ASB2β-Induced Changes to the Skeletal Muscle Proteome and Ubiquitinome

**Fig. 2:**
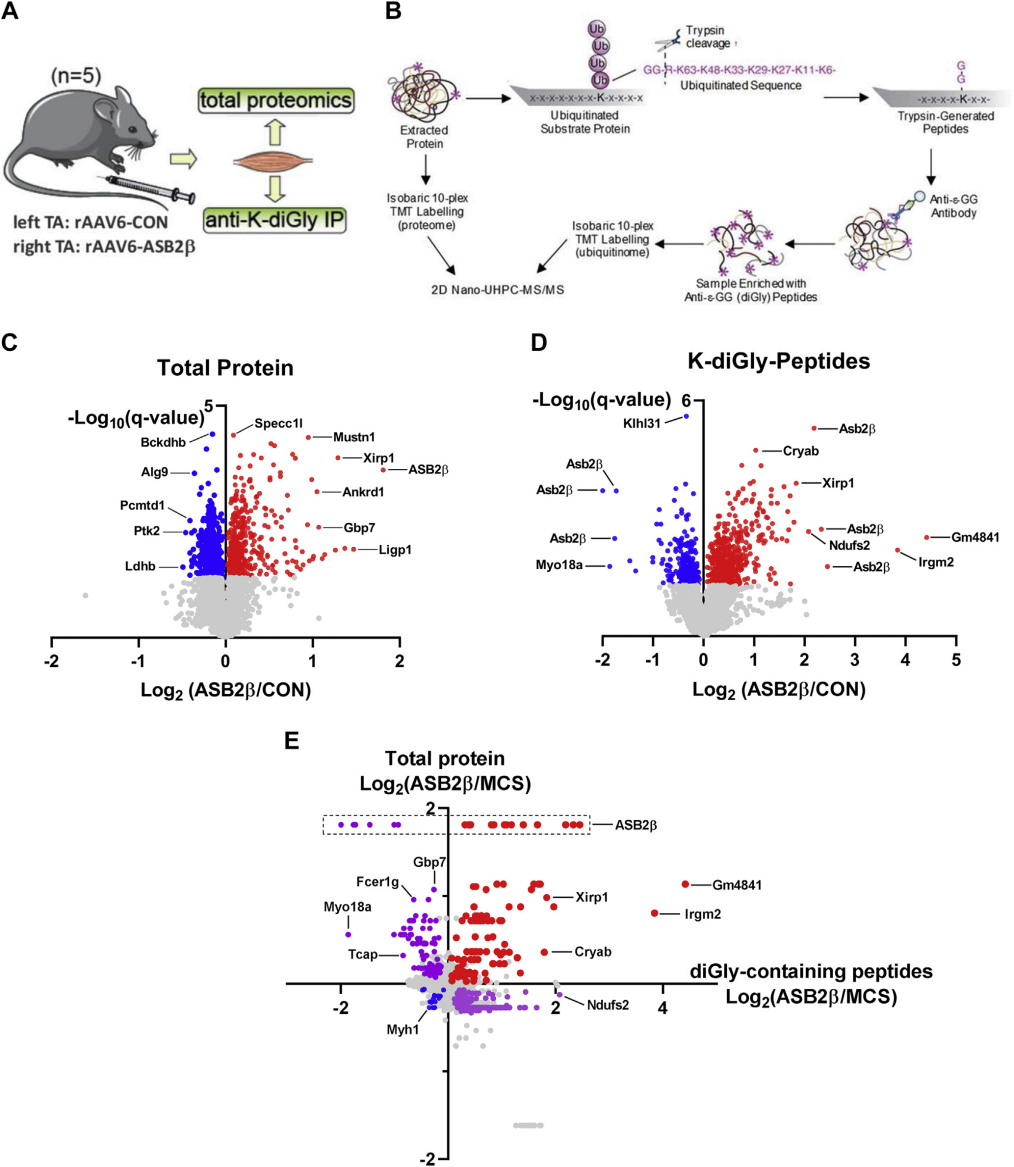
**Quantification of the ASB2β-regulated proteome and ubiquitinome in mouse skeletal muscle.** An overview of the (*A*) experimental design and (*B*) proteomic/ubiquitinomic workflow. Volcano plots showing the Log_2_ fold-change (ASB2β/CON) plotted against the −Log_10_*p* value highlighting significantly regulated (*C*) peptides and (*D*) lysine (K) diGly-modified peptides (*blue* = decreased, *red* = increased; *q* < 0.05, n = 5, *t* test with Benjamini Hochberg FDR). *E*, the correlation between the Log_2_ fold-change (ASB2β/CON) peptide lysine (k) diGly modification and the Log_2_ fold-change (ASB2β/CON) for peptide abundance (*blue* = decreased peptide diGly modification and decreased peptide abundance, *red* = increased peptide diGly modification and increased peptide abundance, *purple* = decreased peptide diGly modification and increased peptide abundance or increased peptide diGly modification and decreased peptide abundance).

To gain insights into how ASB2β might regulate skeletal muscle structure and function, we next performed a quantitative analysis of the relative changes to the proteome and ubiquitinome of mouse limb muscles after an intramuscular injection of rAAV:ASB2β or control vector (rAAV:CON, Fig. 2*A*), as previously described (43). These experiments focused on the time point of 10 days postrAAV injection to detect changes relatively early in the atrophic process (as indicated in Fig. 1*A*). Muscle proteins were extracted and digested with trypsin, which generates a diglycine (diGly) tag covalently attached to lysine residues that represents a remnant of ubiquitinated substrates. It should be noted that the diGly signature does not discriminate between the different types of ubiquitination events (*i.e.*, monoubiquitination *versus* polyubiquitination or branched *versus* linear polyubiquitin chains). Enrichment of diGly-containing peptides was performed with anti-diGly immunoprecipitation (33). Enriched peptides were labeled with isobaric 10-plex TMT and analyzed by 2D nano-UHPLC-MS/MS, including the analysis of peptides without immunoprecipitation to perform a relative quantification of the proteome (Fig. 2*B*). It should be noted that we acquired both diGly and total proteomic data with MS2-based acquisitions and, therefore, ratio compression may reduce detection accuracy and subsequent normalization to total protein abundance. For the proteomic analysis, a total of 4676 protein groups were quantified (3358 with at least two peptides) with a median coefficient of variation of 7.95% and Pearson correlation of 0.99 between the five biological replicates (supplemental Fig. S3). A total of 907 proteins were significantly altered following ASB2β overexpression (q < 0.05, *t*-test with Benjamini–Hochberg FDR; Fig. 2*C*) (supplemental Table S1). Of the differentially abundant proteins associated with increased ASB2β expression, the relative abundance of 459 proteins decreased, while 448 proteins increased (Fig. 2*C*). For the parallel ubiquitinomic analysis, a total of 5551 unique peptides modified with diGly were quantified on 1521 proteins (supplemental Table S2). A change in the abundance of a diGly peptides can arise from either a change in ubiquitination or a change in the abundance of the protein itself. Therefore, we normalized the relative abundance of the diGly peptides to their total protein changes. Out of the 5551 diGly peptides, 1075 diGly peptides were differentially regulated by ASB2β prior to normalization (supplemental Table S2). We were able to normalize 5209 diGly peptides to total protein, with 863 being significantly regulated following ASB2β overexpression (q < 0.05; *t*-test with Benjamini–Hochberg FDR; Fig. 2*D*). Of these 863 differentially regulated diGly peptides, 550 of these remained differentially regulated after normalization, while 313 diGly peptides that were not significantly differentially regulated prior to normalization were significantly regulated after normalization to total protein (supplemental Table S2). These regulated diGly peptides were identified as belonging to 155 proteins that contained lysine residues that only underwent a significant increase in diGly modification, 110 proteins containing lysine residues that only had a significant decrease in diGly modification, and 32 proteins that exhibited both increased and decreased diGly modification on separate lysine residues. Figure 2*E* shows the relationship between the change in peptide ubiquitination and abundance, demonstrating that there is no simple relationship between these two parameters at this time point (*i.e.*, 10 days) and highlighting that an increase in ubiquitination does not necessarily lead to a reduced abundance of the protein. Combined, these data demonstrate that ASB2β-induced muscle atrophy and contractile dysfunction are associated with a dynamic regulation of both the proteome and ubiquitinome.

### Proteins Upregulated by ASB2β

**Fig. 3:**
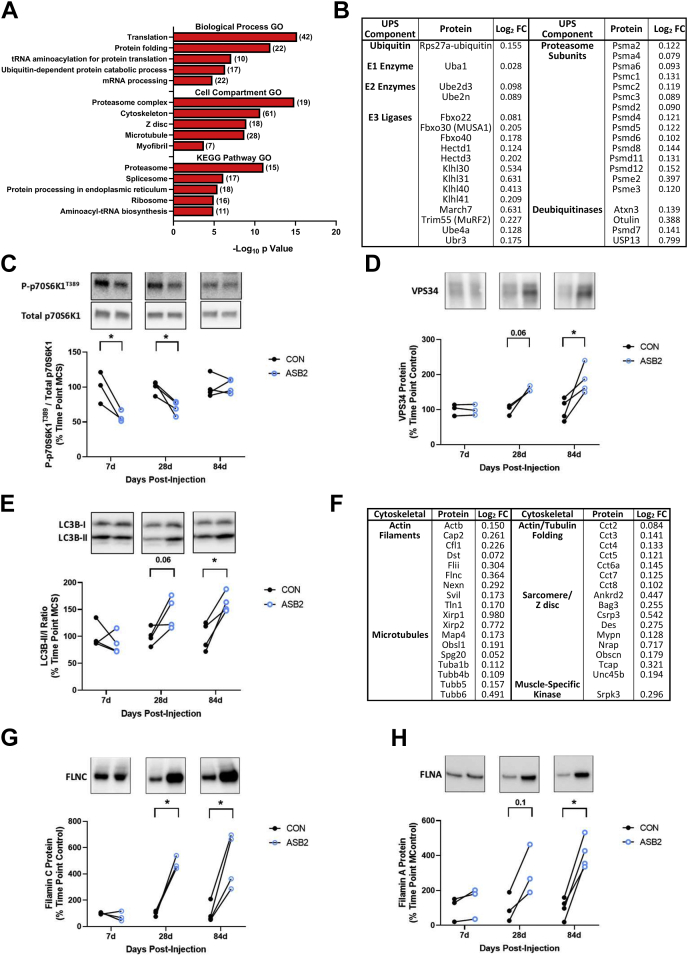
**Pathway enrichment analysis and identification of key proteins upregulated by ASB2β *in vivo*.***A*, database for Annotation, Visualization, and Integrated Discovery (DAVID)-based pathway enrichment gene ontology (GO) analysis of proteins upregulated by 10 days of ASB2β overexpression (in *brackets* are the number of proteins included in each GO term) (n = 5/group). Specific ubiquitin proteasome system-related (*B*) and cytoskeletal/sarcomere-related (*F*) proteins upregulated by ASB2β and their Log_2_ fold change (Log_2_FC). ASB2β-induced changes in p70^S6K1^ T389 phosphorylation (*C*), VPS34 protein (*D*), the LC3BII to LC3BI ratio (*E*), total filamin C protein (*G*) and total filamin A protein (*H*) at 7, 28 and 84 days postinjection. (n = 3–4/group). ∗*p* < 0.05.

To identify biological processes potentially affected by increased expression of ASB2β in skeletal muscle, we conducted a gene ontology (GO) analysis (DAVID Bioinformatic Database, https://david.ncifcrf.gov/) of the proteomic data obtained from muscles administered AAV:ASB2β or AAV:Con (Fig. 3*A*). Analysis of proteins upregulated by ASB2β identified several GO terms related to ubiquitin-dependent protein degradation and the proteasome. Consistent with these terms, ASB2β increased the expression of ubiquitin (Rps27a is a fusion protein comprising ubiquitin and Rps27a, which is cleaved to release monomeric ubiquitin), E1 and E2 enzymes, E3 ligases, subunits of the proteasome, and DUBs (Fig. 3*B*). Included among the upregulated E3 ligases are MUSA1 (Fbxo30), the muscle-specific Fbxo40 and MuRF2 (Trim55), and Kelch superfamily proteins, Klhl31 and Klhl41, both of which play roles in regulating muscle structure and function (44, 45, 46). Furthermore, another non-UPS protease that was identified as upregulated by ASB2β was the cysteine-aspartic protease, caspase 3 (Casp3; Log_2_ FC 0.636, supplemental Table S1). These data suggest that ASB2β-induced muscle atrophy is, in part, mediated by activation of the UPS and subsequent protein degradation.

The biological process GO terms also identified proteins involved in translation (*i.e.*, protein synthesis) as being upregulated by ASB2β. These proteins include translation initiation and elongation factors, ribosomal proteins, and tRNA synthetases that charge specific tRNAs with their respective amino acids (supplemental Fig. S4). While these results suggest that there may have been an increase in protein synthesis, because ASB2β expressing muscles underwent muscle atrophy, any increase in the rate of synthesis must have been insufficient to counter the increase in protein degradation. Protein synthesis in muscle is regulated by a range of factors, including activation by the mechanistic target of rapamycin complex 1 (mTORC1) (1, 47). Furthermore, mTORC1 is known to regulate the translation of mRNAs encoding numerous translation factors and ribosomal proteins, including many of those regulated here by ASB2β (48). As such, using western blotting, we examined whether ASB2β had activated mTORC1 signaling by examining for an increase in the phosphorylation of the T389 residue of the direct mTORC1 target, p70^S6k1^ (49). However, T389 phosphorylation, relative to total p70^S6k1^ protein, was in fact decreased in ASB2β expressing muscles between at 7 and 28 days postinjection (Fig. 3*C*). This finding suggests that the ASB2β-induced increase in proteins involved in translation/protein synthesis likely occurred *via* an mTORC1-independent mechanism.

In addition to positively regulating protein synthesis, mTORC1 is also an inhibitor of autophagy (50). Interestingly, the proteomic analysis identified several autophagy-related proteins as being upregulated at 10 days by ASB2β (*i.e.*, Ankrd13a, Bag3, Hspa8, Optn, Sqstm1/p62, Ubxn4, Ufd1l, and Vcp; supplemental Table S1), including the DUB, Usp13, which has been shown to deubiquitinate and stabilize the class III phosphoinositide 3-kinase, vacuolar protein sorting 34 (Vps34), to promote autophagy initiation (51). Indeed, we found that Vps34 was elevated at 84 days after vector administration, with a strong trend (*p* = 0.06) to be elevated at 24 days (Fig. 3*D*). Combined, these data suggest that ASB2β-induced muscle atrophy may also be associated with the activation of autophagy-mediated protein degradation, in part, *via* the suppression of mTORC1 signaling and elevated Vps34. To examine this hypothesis, we analyzed the LC3B-II/LC3B-I ratio, a marker of autophagosome formation (52), and found that, similar to VPS34 expression, this ratio was significantly increased at 84 days of ASB2β expression, with a strong trend to be elevated (*p* = 0.06) at 28 days (Fig. 3*E*). Given that mTORC1 signaling was normalized by 84 days postinjection, these data suggest that ASB2β-induced autophagy may be driven by both mTORC1-dependent and mTORC1-independent mechanisms.

Other major GO terms associated with proteins upregulated by ASB2β are related to structural proteins of the cytoskeleton and the organization of the sarcomere/Z disc. These proteins include beta actin (Actb), tubulin isoforms, members of the actin/tubulin chaperonin containing tailless complex polypeptide 1 (CCT) molecular chaperone family (53), and actin filament and microtubule binding/interacting proteins, such as the nonmuscle F-actin depolymerization factor, colfilin 1 (Cfl1), and the actin stabilizing factors, Xin actin-binding repeat proteins 1 & 2 [Xirp1 & 2; (54)] (Fig. 3*F*). Surprisingly, the putative ASB2 target (55), filamin C (Flnc), was also identified as being upregulated by ASB2β (Fig. 3*G*). To determine whether this was a transient event, or a more prolonged adaptation, we investigated Flnc expression using western blotting and found that Flnc abundance was unchanged at 7 days but remained markedly elevated at 28 days and 84 days (Fig. 3*G*). Furthermore, although not identified as differentially expressed in the proteomics analysis, our western blot analysis determined that filamin A (Flna) was also elevated at 84 days with a trend for an increase at 28 days (Fig. 3*H*). Interestingly, the proteomic analysis also identified filamin A-interacting protein 1 (FILIP1), which is known to induce the degradation of Flna and Flnc (56, 57), as being upregulated (Log_2_ FC - 0.210, supplemental Table S1), potentially in response to the increased presence of Flna and Flnc. Several sarcomere/Z disc proteins were also upregulated, including telethonin (Tcap), ankyrin repeat domain 2 (Ankrd2), myopalladin (Mynp), nebulin-related-anchoring protein (Nrap), the myosin chaperone, Unc45, which plays an essential role in myofibrillogenesis (58), and the intermediate filament protein, desmin (Des) (Fig. 3*F*). There was also an upregulation of the muscle-specific kinase, Srpk3, whose overexpression has been shown to cause muscle degeneration (59). The GO analyses also identified that proteins involved in the biological processes of mRNA processing and spliceosome function were upregulated in ASB2β overexpressing muscles, suggesting a possible role for ASB2β in the nucleus and the regulation of mRNA processing. Overall, the proteomic analysis of upregulated proteins reveals that ASB2β-induced muscle atrophy is associated with activation of UPS- and autophagy-mediated protein degradation, a potential increase in translation, and what appears to be extensive cytoskeletal and sarcomere remodeling as the muscles undergo a reduction in mass.

### Proteins Downregulated by ASB2β

**Fig. 4:**
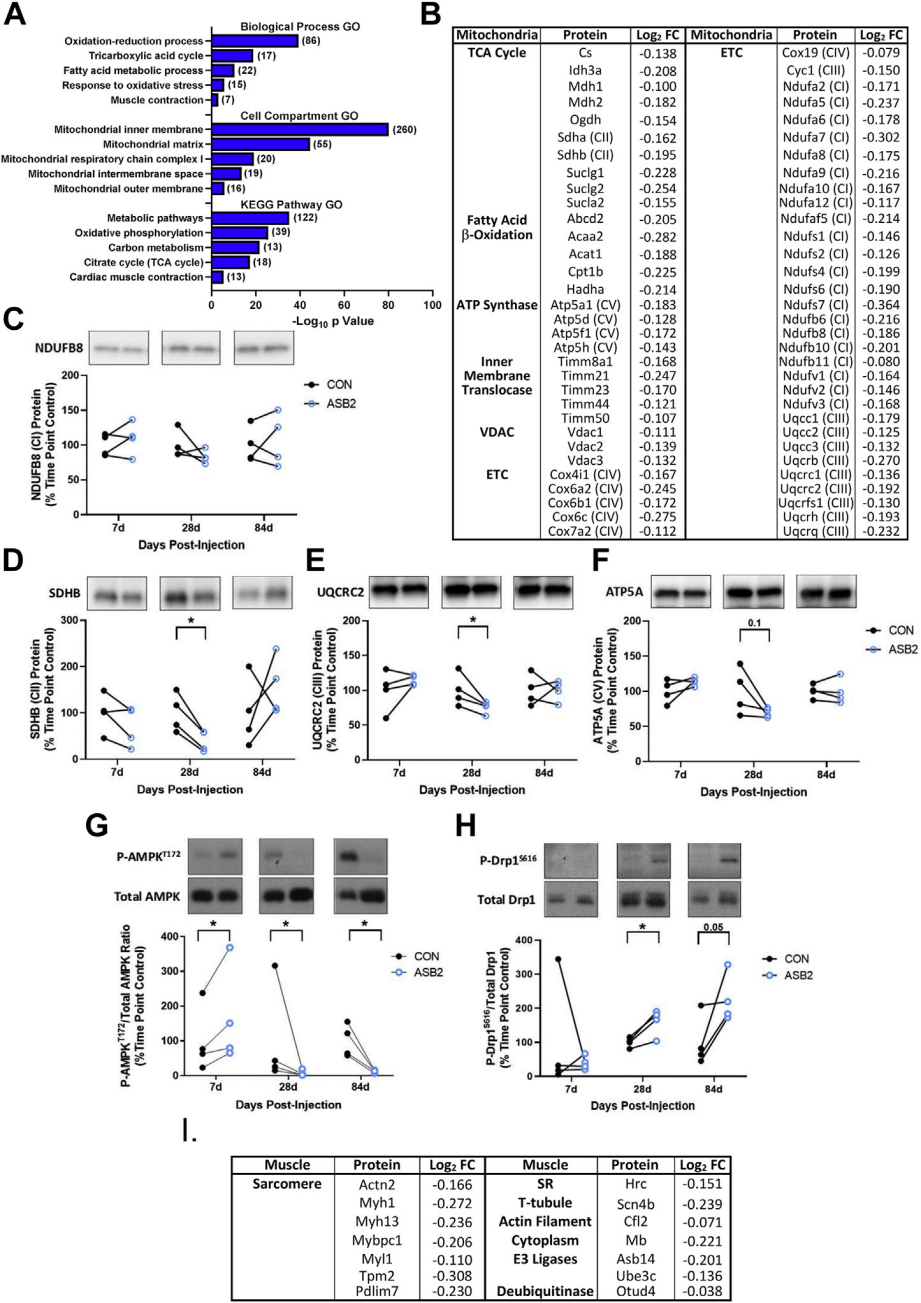
**Pathway enrichment analysis and identification of key proteins downregulated by ASB2β *in vivo*.***A*, database for Annotation, Visualization, and Integrated Discovery (DAVID)-based pathway enrichment gene ontology (GO) analysis of proteins downregulated by 10 days of ASB2β overexpression (in *brackets* are the number of proteins included in each GO term) (n = 5/group). Specific mitochondrial-related (*B*) and muscle-related (*I*) proteins downregulated by ASB2β and their Log_2_ fold change (Log_2_FC) (TCA cycle, tricarboxylic acid cycle; VDAC, voltage-dependent anion channel; ETC, electron transport chain). ASB2β-induced changes in total NDUFB8 (CI) protein (*C*), total SDHB (CII) protein (*D*), total UQCRC2 (CIII) protein (*E*), total ATP5A (CV) protein (*F*), AMPK T172 phosphorylation (*G*) and Drp1 S616 phosphorylation (*H*) at 7, 28, and 84 days postinjection (n = 4/group). ∗*p* < 0.05.

Regarding proteins that were downregulated by ASB2β, mitochondrial or mitochondria-associated, proteins were particularly abundant (supplemental Table S1). The GO analysis of the downregulated proteins identified several terms related to mitochondrial function and metabolism that were significantly overrepresented, including oxidation–reduction process, tricarboxylic acid (TCA) cycle, and respiratory electron transport chain (ETC), while several mitochondrial compartments were in the top cellular compartment GO terms (Fig. 4*A*). Biological processes downregulated by ASB2β included specific fatty acid β-oxidation, TCA cycle, outer membrane voltage-dependent ion channel (VDAC), inner membrane translocases (Timms), and proteins from all five of the ETC complexes (CI, II, III, IV, and V) (Fig. 4*B*). To determine whether the decrease in mitochondrial proteins had already occurred by 7 days and/or was maintained up to 84 days, we used western blotting combined with an antibody cocktail against select ETC proteins that were also identified as decreased by ASB2β in the 10 days proteomics data [*i.e.*, NDUFB8 (CI), SDHB (CII), UQCRC2 (CIII) and ATP5A (CV); Fig. 4*B*]. This analysis found that at 28 days, SDHB (CII; Fig. 4*D*) and UQCRC2 (CIII; Fig. 4*E*) were significantly reduced as a consequence of increased ASB2β expression, with a trend for ATP5A (Fig. 4*F*) to also be lower at 28 days, but there was no change in the abundance of these proteins at 7 or 84 days (Fig. 4, *C*–*F*). Collectively, these data suggest that the ASB2β-induced reduction in mitochondrial proteins is a dynamic event, commencing between 7 and 10 days and returning to normal by 84 days. Moreover, these data suggest that an early ASB2β-induced event may have triggered the transient decrease in mitochondrial protein expression or a reduction in mitochondrial number.

A decrease in mitochondrial protein could lead to bioenergetic stress within the muscle, and one enzyme that is sensitive to changes in cellular energy status is AMP-activated protein kinase (AMPK). AMPK is activated, in part, by a decrease in the ATP/AMP ratio, as indicated by an increase in the T172 phosphorylation of the AMPK α-subunit (60, 61). Thus, we used western blotting to investigate whether ASB2β expression may have led to changes to AMPK T172 phosphorylation and found that AMPK phosphorylation was significantly elevated at 7 days (Fig. 4*G*); however, by 28 days, this activation was reversed to a marked inhibition that continued to 84 days. Furthermore, there appeared to be an increase in total AMPK protein at 28 and 84 days (Fig. 4*G*), which was confirmed in the 10-day proteomic data with an increased abundance of the AMPK α2 subunit (Prkaa2; supplemental Table S1). These data show that increased ASB2β expression led to a transient early activation of AMPK prior to the first detection of a decrease in mitochondrial proteins in the proteomic analysis at 10 days.

A disruption in the normal balance between mitochondrial fission and fusion can lead to the activation of proteasome- and autophagy-mediated protein degradation and muscle atrophy [for recent review see (62)]. In this context, it is interesting to note that our 10-day proteomic analysis identified a significant ASB2β-induced decrease in the abundance of the mitochondrial fusion-promoting, Optic Atrophy Protein 1 (Opa1; Log_2_ FC −0.140; supplemental Table S1) (63), potentially contributing to a shift in the balance toward mitochondrial fission. AMPK activation is sufficient to induce a range of cellular processes, including mitochondrial fission (64), while mitochondrial dysfunction can also lead to reversible mitochondrial fission that is dependent on the state of actin polymerization (65, 66, 67, 68). Recent studies have suggested that the nonmuscle F-actin depolymerization factor, cofilin (Cfl1), and cytoskeletal protein, Flna, play roles in the mitochondrial fission process (65, 69, 70), and we have identified both of these proteins as being upregulated by ASB2β. Mitochondrial fission is, in part, regulated by the GTPase, dynamin-related protein 1 (Drp1), with the phosphorylation of the S616 residue in Drp1 stimulating mitochondrial fission (71, 72, 73). Therefore, we examined whether this ASB2β-induced cytoskeletal remodeling might be associated with Drp1 S616 phosphorylation. Indeed, using western blotting, we found increased phosphorylation of this site at 28 and 84 days, with no significant change in total Drp1 protein (Fig. 4*H*). These data suggest the possibility of an ASB2β-induced disruption of the balance between mitochondrial fission and fusion that is preceded by AMPK activation and associated with cytoskeletal remodeling and a transient reduction in mitochondrial protein.

In addition to the ASB2β-induced reduction in mitochondrial proteins, the GO analysis of our proteomic data also identified the biological process term of muscle contraction, and the cell compartment term of myosin complex, as being significantly overrepresented. Specifically, the abundance of several contractile protein isoforms was reduced, including fast myosin light chain 1 (Myl1) and type 2 days/x fast myosin heavy chain (Myh1, Fig. 4*I*). Proteins involved in sarcomere organization [myosin-binding protein C1 (Mybpc1) and alpha actinin 2 (Actn2)], sarcoplasmic reticulum Ca^2+^ storage [sarcoplasmic reticulum histidine-rich calcium-binding protein (Hrc)], T-tubule voltage sensing [sodium voltage-gated channel beta subunit 4 (Scn4b)], and the muscle-specific isoform of the F-actin depolymerization factor, cofilin [Cfl2; (74)], were also decreased (Fig. 4*I*). Three other notable proteins that were reduced by ASB2β were myoglobin (Mb) and the E3 ligases, Ube3c and Asb14. While very little is known about the function of Asb14, its expression has been shown to be highly enriched in skeletal muscle (https://www.gtexportal.org/home/gene/ASB14). The reductions in the abundance of these excitation–contraction coupling, contractile, and sarcomere/cytoskeletal proteins have the potential to, at least in part, explain the ASB2β-induced impairment in specific force production.

### ASB2β-Induced Alterations to the Ubiquitinome

**Fig. 5:**
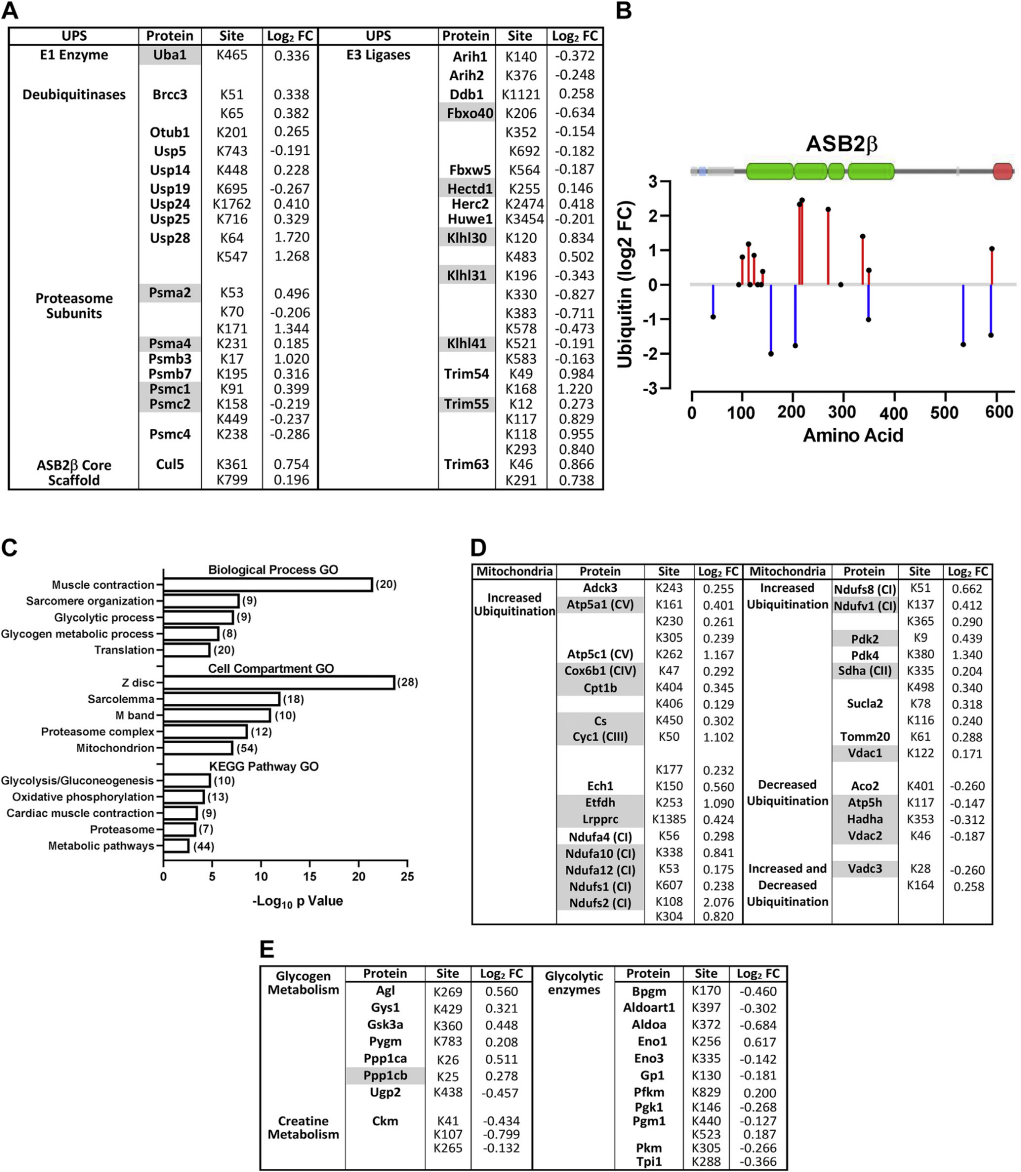
**Analysis of proteins identified as having a relative ASB2β-induced change in ubiquitination status *in vivo*.***A*, list of ubiquitin proteasome system (UPS)-related proteins and specific lysine (K) residues with altered ubiquitination with ASB2β overexpression and the relative Log_2_ fold change (Log_2_FC) (Note: proteins highlighted in gray were identified as also having an increase in their total abundance; see Fig. 3 and supplemental Table S1). *B*, changes to the ubiquitination status of specific ASB2β lysine residues with the corresponding major domain structure of ASB2β superimposed onto the graph (obtained from VisQuant (https://visquant.cmri.org.au/ using UniProt ASB2β ID# Q8K0L0)). (*Black dots* on the X-axis represent lysine residues that were identified as being ubiquitinated but did not undergo a statistically significant change in their ubiquitination status with ASB2β overexpression compared with the control condition, CON). *C*, database for Annotation, Visualization, and Integrated Discovery (DAVID)-based pathway enrichment gene ontology (GO) analysis of proteins with altered ubiquitination by 10 days of ASB2β overexpression (in *brackets* are the number of proteins included in each GO term) (n = 5/group). *D*, specific mitochondrial proteins and (*E*) glycogen metabolism-related proteins and glycolytic enzymes that underwent a significant change in ubiquitination by ASB2β and the Log_2_ fold change (Log_2_FC) in ubiquitination at specific lysine (K) residues (Note: proteins highlighted in *gray* were identified as also having an increase in their total abundance; see Fig. 3 and supplemental Table S1).

As expected, increased ASB2β expression also induced extensive changes to the muscle ubiquitinome. Depending on the exact site and the length (mono-*versus* poly-ubiquitination) and type of ubiquitin chain (linear *versus* branched), these changes in the ubiquitinome will likely play a role in targeting some proteins for degradation by the proteome, while for other proteins, altered ubiquitination will lead to changes in protein function, localization, trafficking, protein–protein interactions, and signaling (75). It should be noted, however, that the changes to the ubiquitinome are unlikely to be mediated solely by ASB2β, but may also be related, in part, to our finding of increased expression of several other E3 ligases and a DUB (see Fig. 3*B*). In addition to other posttranslational modifications (*e.g.*, phosphorylation), E3 ligase and DUB activity may also be regulated by their own state of ubiquitination (76, 77, 78) and, as such, we examined our data set for significant changes in the diGly signature on E3 ligases and DUBs and other UPS-related proteins. There were 14 E3 ligases and eight DUBs that underwent significant changes in their ubiquitination status on single or multiple sites (Fig. 5*A*), including some of the E3 ligases whose expression was upregulated by ASB2β (identified in gray in Fig. 5*A*). In addition, we identified the E1 enzyme, Uba1, and several of the proteasome subunits, as also undergoing a significant alteration to their ubiquitination status (Fig. 5*A*). Furthermore, Cul5, the core scaffold protein of several E3 ligase complexes (79), including ASB2β, also underwent an increase in ubiquitination at two lysine residues (Fig. 5*A*). Finally, although the level of ASB2β was experimentally elevated, we also examined changes to its ubiquitination status and graphed these relative to the lysine residue number and superimposed ASB2β’s major domain structure obtained from VisQuant (https://visquant.cmri.org.au/; using UniProt ID# Q8K0L0). ASB2β underwent the most extensive alteration in its ubiquitination status compared with all other identified E3 ligases, with increases and decreases in the ubiquitination of multiple lysine residues across almost the entire length of the protein (Fig. 5*B*). The modifications include reduced ubiquitination (K42) in the N-terminal UIM motif (residues 26–45) and opposing changes to ubiquitination on two residues (K586 & K588) in the C-terminal SOCS box domain (residues 580–634). Combined, these data show that key proteins involved in the UPS not only regulate the ubiquitination other cellular proteins but are themselves subject to ubiquitination.

In regard to changes in the broader muscle ubiquitinome, cell compartment GO term analysis of all proteins that underwent a significant change (increase or decrease) in ubiquitination revealed an overrepresentation of proteins from the mitochondria (Fig. 5*C*), with the majority of these proteins undergoing an increase in ubiquitination, including several from complex I of the ETC (Fig. 5*D*). Moreover, many of the mitochondrial proteins with increased ubiquitination were also observed to exhibit a reduction in total abundance (highlighted in gray). These results suggest the possibility that the reduction in the amount of these proteins may be related to the increase in ubiquitination; however, there were also a few proteins that underwent a decrease in abundance even though they experienced a reduction in ubiquitination or demonstrated both an increase and a decrease in ubiquitination on separate lysine residues (Fig. 5*D*). These results highlight the complexity of the regulation of individual protein turnover and suggest that other factors likely also play a role. Nonetheless, these data suggest a positive correlation between the change in abundance and the ubiquitination status for some critical mitochondrial proteins and provide insight into how the early effect of ASB2β could influence mitochondrial function/number.

In the context of the effect of ASB2β on the ubiquitination of proteins involved in energy production, it is interesting to note that the biological process GO term analyses also identified glycolytic and glycogen metabolic processes as being overrepresented. More specifically, six out of seven glycogen metabolism-related proteins, including glycogen phosphorylase (Pygm) and glycogen synthase (Gys1), exhibited an increase in ubiquitination (Fig. 5*E*), while nine out of 11 glycolytic enzyme proteins demonstrated a decrease in ubiquitination, with one glycolytic protein (phosphoglucomutase 1, Pgm1) presenting an increase and decrease in ubiquitination on separate lysine residues. Furthermore, of all these proteins, only one exhibited a change in total abundance (*i.e.*, an increase in protein phosphatase 1 catalytic subunit beta, Ppp1cb). Another important protein associated with energy metabolism is the muscle isoform of creatine kinase (CKM), which, similar to the aforementioned glycolytic enzymes, exhibited a decrease in ubiquitination at three lysine residues. While it is not immediately clear why there is such a stark difference in the direction of the change in ubiquitination for these groups of metabolic proteins in the absence of any substantial change in total protein, the data present the possibility that ubiquitination could contribute to the regulation of metabolic enzyme function during ASB2β-induced muscle atrophy.

**Fig. 6:**
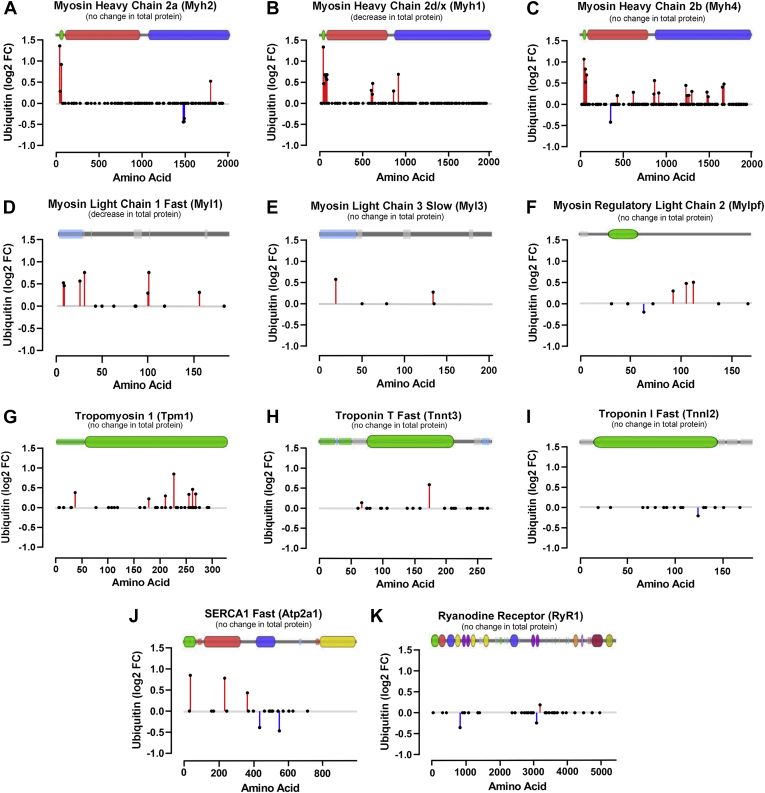
**ASB2β overexpression-induced changes in the *in vivo* ubiquitination status of specific contractile and excitation–contraction coupling proteins.** Changes to the ubiquitination status of specific lysine residues with the corresponding major domain structure of each protein is superimposed onto each graph (obtained from VisQuant (https://visquant.cmri.org.au/ using UniProt mouse protein ID numbers)). *A*, myosin heavy chain 2a (Myh2, Q9WUS5), *B*, myosin heavy chain 2 days/x (Myh1, Q5SX40), *C*, myosin heavy chain 2b (Myh4, Q5SX30), *D*, myosin light chain 1 (Myl1, P05977), *E*, myosin light chain 3 (Myl3, P09542), *F*, myosin regulatory light chain 2 (Mflpf, P97457), *G*, tropomyosin 1 (TPM1, F8WID5), *H*, troponin T fast (Tnnt3, Q9QZ47), *I*, troponin I fast (TnnI2, P13412), *J*, Serca1 Fast (ATP2a1, Q8R429) and *K*, ryanodine Receptor 1 (RyR1, E9PZQ0). (*Black dots* on the X-axis represent lysine residues that were identified as being ubiquitinated but did not undergo a statistically significant change in their ubiquitination status with ASB2β overexpression compared with the control condition, CON. See supplemental Table S2).

The most highly overrepresented biological process and cell compartment GO terms for proteins with altered ubiquitination status were Muscle Contraction and the Z Disc, respectively. The TA muscle from the C57Bl/6 mouse strain is predominantly composed of fast-twitch muscle fiber types, 2A (∼6%), 2D/X (∼30–35%), and 2B (∼60%) (80), and all three fast myosin heavy chain (MyHC) isoform proteins that correspond with these fiber types [2a (Myh2), 2 days/x (Myh1) and 2b (Myh4), respectively] exhibited changes in ubiquitination status with ASB2β overexpression (Fig. 6, *A*–*C*). While several lysine residues along the length of each protein underwent a change (increase or decrease) in their ubiquitination status, all three MyHC isoforms possess a cluster of lysines located in the far N-terminal Src homology 3 (SH3)-like subdomain (residues 33–82, green region in Fig. 6, *A*–*C*), and in particular K44, which presented the greatest increase in ubiquitination. This N-terminal head region of the myosin filament is essential for its function, and the SH3-like subdomain has been proposed to interact with essential/alkali myosin light chains (MLC) to modulate myosin kinetics (81, 82). Thus, increased ubiquitination in this subdomain has the potential to influence protein-to-protein interaction and, therefore, muscle contractility. In this context, it is of interest to note that three MLC were also detected as having altered ubiquitination (Fig. 6, *D*–*F*), including the essential MLC, fast MLC-1 (Myh1), which had the most extensive change in ubiquitination and whose abundance was decreased by ASB2β. Other critical contractile and E-C coupling proteins were also detected as having a significant change in ubiquitination (but no change in total abundance), including the fast isoforms of tropomyosin (Tpm1), troponin T (Tnnt3) and troponin I (TnnI2) and the fast isoform of the sarcoplasmic reticulum (SR) Ca^2+^-ATPase, SERCA 1 (ATP2a1), and the SR Ca^2+^ release channel, ryanodine receptor 1 (RyR1) (Fig. 6, *G*–*K*). Overall, these findings suggest the possibility that ASB2β-induced changes to ubiquitination of contraction-related proteins may play a role in modulating their abundance and/or function.

**Fig. 7:**
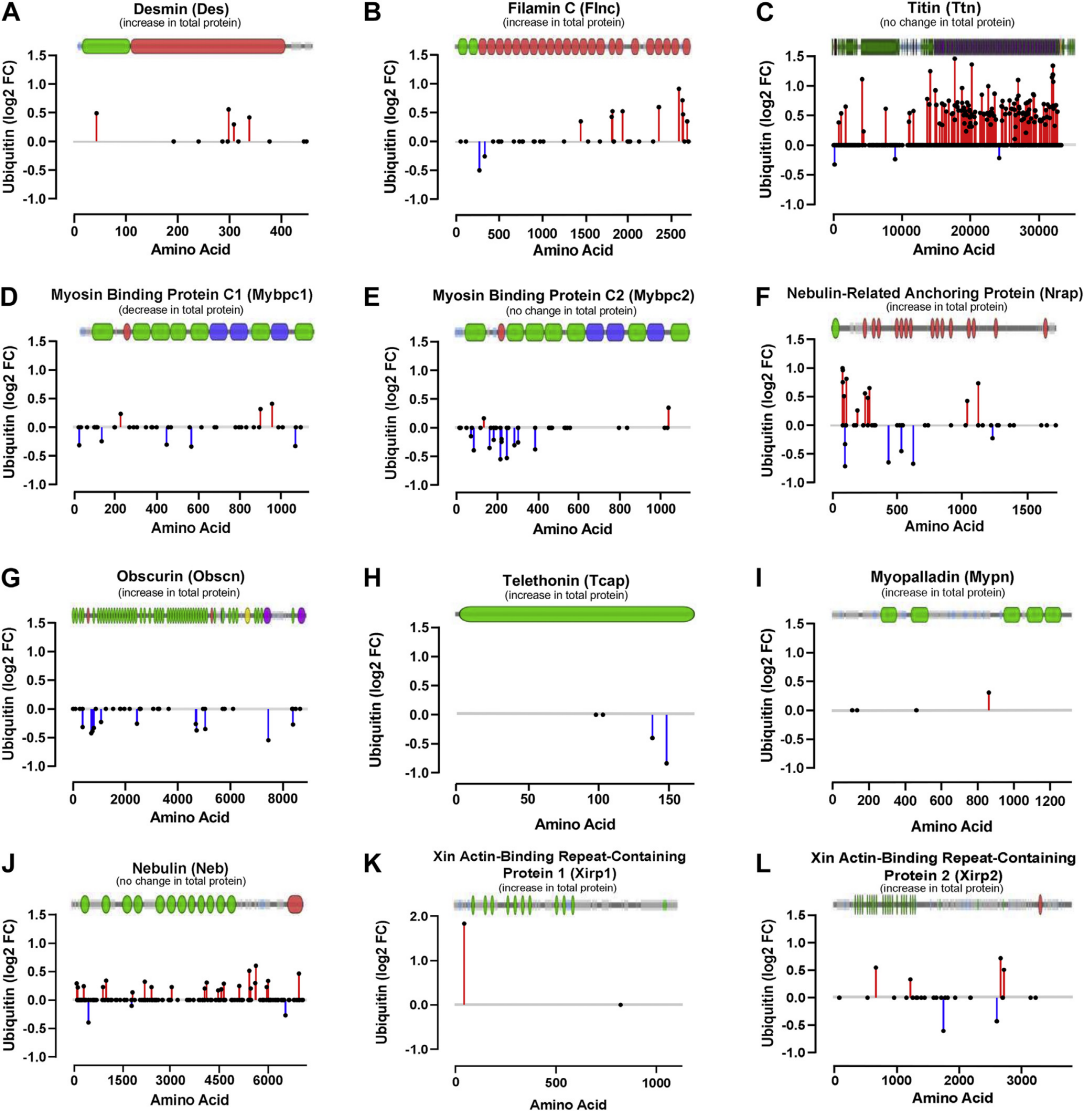
**ASB2β overexpression-induced changes in the *in vivo* ubiquitination status of specific cytoskeletal and sarcomeric proteins.** Changes to the ubiquitination status of specific lysine residues with the corresponding major domain structure of each protein are superimposed onto each graph (obtained from VisQuant (https://visquant.cmri.org.au/ using UniProt mouse protein ID numbers)). *A*, Desmin (Des, P31001), *B*, Filamin C (Flnc, Q8VHX6), *C*, Titin (Ttn, A2ASS6), *D*, myosin binding protein C1 (Mybpc1,Q6P6L5), *E*, myosin binding protein C2 (Mybpc2, Q5XKE0), *F*, nebulin-related anchoring protein (Nrap, Q80XB4), *G*, obscurin (Obscn, A2AAJ9), *H*, telethonin (Tcap, O70548), *I*, myopalladin (Mypn, Q5DTJ9), *J*, nebulin (Neb, E9Q1W3), *K*, Xin actin-binding repeat-containing protein 1 (Xirp1, O70373) and *L*, Xin actin-binding repeat-containing protein 2 (Xirp2, Q4U4S6). (*Black dots* on the X-axis represent lysine residues that were identified as being ubiquitinated but did not undergo a statistically significant change in their ubiquitination status with ASB2β overexpression compared with the control condition, CON. See supplemental Table S2).

As ASB2β has been shown to colocalize at the Z disc and induce the degradation of the intermediate filament protein, desmin, in cardiac muscle cells (21), we had anticipated that the ubiquitination status of desmin and other Z-disc, or Z-disc-interacting proteins, might be altered by ASB2β. Consistent with this hypothesis, a significant increase in the ubiquitination of desmin lysine residues was detected in the N-terminal head domain (green) and the central rod domain (red, Fig. 7*A*); however, as detailed above, this increased ubiquitination was associated with an increase in the abundance of desmin. Likewise, the skeletal muscle filamin isoform, Flnc, which underwent even more extensive changes to its ubiquitination status than desmin (Fig. 7*B*), also demonstrated an increase in total abundance. As the largest protein in mammalian cells (∼3.7 MDa), titin (Ttn) plays a central role in the structure and function of the sarcomere, influencing passive and active force production and molecular signaling (83). Titin spans the distance between the sarcomeric Z disc and the central M-band and, in part, forms a scaffold to which numerous other contractile, structural, and signaling proteins bind, including the E3 ligases, MuRF1 (Trim63) and MuRF2 (Trim55), toward the extreme C-terminal (M band) end (83). Therefore, in addition to ASB2β, MuRF1, and particularly MuRF2, whose abundance was increased by ASB2β, may play a role in regulating titin ubiquitination during ASB2β-induced muscle atrophy. In muscles overexpressing ASB2β, we detected profound changes in ubiquitination across the entire length of titin (Fig. 7*C*), but especially in the C-terminal domain that is associated with the region of actin and myosin filament overlap (*i.e.*, A-band) and M-band interaction (83). Other proteins that bind to the C-terminal region of titin include the myosin-binding protein C isoforms (Mybpc1 and Mybpc2), the nebulin-related anchoring protein (Nrap), and obscurin (Obscn), all of which underwent significant changes in their ubiquitination status (Fig. 7, *D*–*G*). Interestingly, Nrap, whose total abundance was increased by ASB2β, exhibited both increases and decreases in ubiquitination on numerous lysine residues (Fig. 7*F*), while Obscn, whose abundance was also increased by ASB2β, exhibited only decreased ubiquitination at multiple sites (Fig. 7*G*). For the Mybpc isoforms, for which there was a decrease in total Mybpc1 and no change in total Mybpc2, both these proteins predominantly presented a decrease in ubiquitination (Fig. 7, *D* and *E*). Proteins that bind near the opposing N-terminal region of titin include the titin capping protein, telethonin (Tcap), which has been shown to also form a complex with MuRF1 E3 ligase and UPS E2 enzymes (84), myopalladin (Mypn), and the large scaffold protein, nebulin (Neb). For Tcap, whose expression was increased by ASB2β, there was reduced ubiquitination on two C-terminal lysine residues (Fig. 7*H*), while Mypn, whose abundance was also increased, presented an increase in ubiquitination on only one lysine residue (Fig. 7*I*). Conversely, Neb, whose abundance was not altered, exhibited predominantly increases in ubiquitination across the entire length of the protein (Fig. 7*J*). Finally, while both xin actin-binding repeat-containing proteins isoforms 1 and 2 (Xirp1 and 2) increased in abundance, Xirp1 exhibited a large increase in ubiquitination at a single N-terminal lysine residue (K44), and Xirp2 demonstrated increases and decreases in the ubiquitination status of several lysine residues (Fig. 7, *K* and *L*). When combined, these data show that ASB2β-induced muscle atrophy is associated with changes in the ubiquitination status of numerous contractile and sarcomeric proteins and that there is no simple relationship between the magnitude and direction of the change in ubiquitination and the change in the abundance of a given protein.

### The ASB2 Interactome and Putative ASB2β Targets

**Fig. 8:**
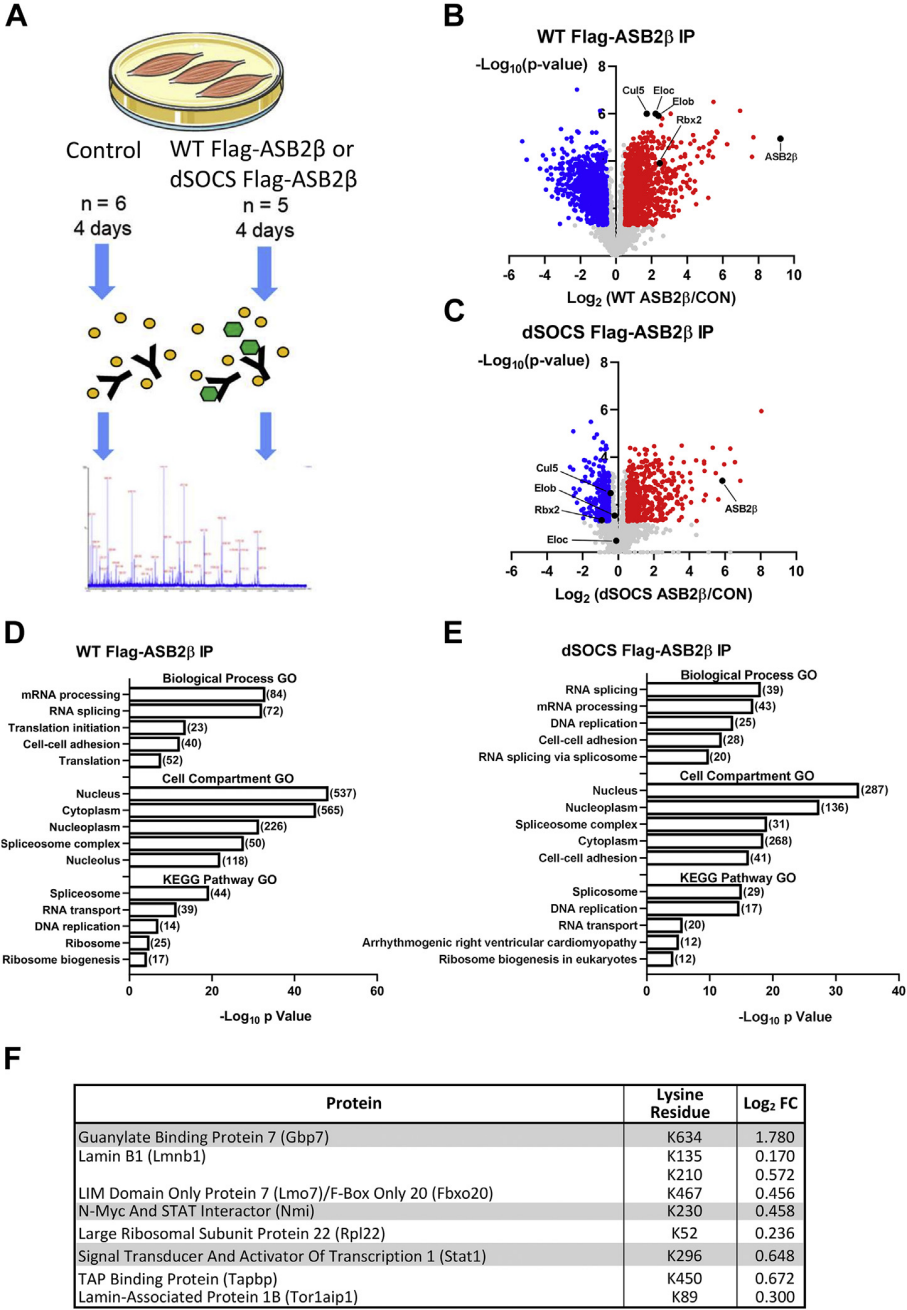
**The ASB2β interactome and putative ASB2β target substrates.***A*, overview of experimental design. C2C12 myoblasts were transfected with DNA plasmid encoding Flag-tagged wild type (WT) ASB2β, a Flag-Tagged mutant ASB2β with the C-terminal SOCS box deleted (dSOCS) or the noncoding control (CON). Commencing 24 h after transfection, myoblasts were differentiated for 4 days, after which, the myotubes were collected and subjected to anti-Flag immunoprecipitations (IP). The enriched samples were then subjected to label-free proteomics to identify ASB2β interacting proteins. Volcano plots showing the Log_2_ fold-change enrichment of proteins associated with (*B*) WT Flag-ASB2β (WT ASB2β/CON) and (*C*) dSOCS Flag-ASB2β (dSOCS ASB2β/CON), plotted against the −Log_10_*p* value (*blue* = decreased enrichment, *red* = increased enrichment; *q* < 0.05 and Log_2_ < −05 & >± 0.5, n = 5–6, *t* test with Benjamini–Hochberg FDR). *C* and *D*, database for Annotation, Visualization, and Integrated Discovery (DAVID)-based pathway enrichment gene ontology (GO) analysis of proteins enrichment with WT ASB2β IP (*D*) and dSOCS ASB2β (*E*) (in brackets are the number of proteins included in each GO term). *F*, to identify a list of putative of ASB2β targets, proteins from C2C12 cells that were pulled down with both Flag-tagged WT ASB2β and Flag-tagged dSOCS ASB2β were compared with proteins from TA muscles *in vivo* that underwent a significant increase in ubiquitination with ASB2β overexpression. The list includes the protein names, the specific lysine (K) residue that underwent a significant ASB2β-induced change in ubiquitination status *in vivo*, and the ASB2β-induced Log_2_ fold change in ubiquitination at that specific site (Note: proteins highlighted in *gray* were identified as also having an increase in their total abundance; see supplemental Table S1).

To gain insights into proteins that interact with ASB2β, and therefore possible ASB2β target substrates, specifically in muscle cells, we transfected C2C12 myoblasts with DNA plasmids encoding Flag-tagged WT ASB2β, a Flag-tagged mutant ASB2β lacking the C-terminal SOCS box domain (dSOCS), or a control plasmid (CON). Twenty-four hours after transfection, myoblast differentiation was initiated and the resulting myotubes were collected 4 days later followed by anti-Flag immunoprecipitation and proteomic analysis (Fig. 8*A*). Because the ASB2β-dSOCS mutant lacks its SOCS box domain, it is unable to bind to its partner scaffold protein, Cul5. Using this strategy, we were able to compare proteins that are associated with the larger ASB2β/Cul5 complex with those associated with ASB2β *per se*.

Immunoprecipitation and subsequent proteomic analysis of proteins from cells expressing WT Flag-ASB2β identified 1106 proteins being significantly enriched compared with cells administered the CON plasmid (q < 0.05 and Log_2_ < −0.5 & >±0.5; Fig. 8*B* and supplemental Table S3). Reassuringly, these included ASB2β, Cul5, Rbx2 (Rnf7), elongin B (Tceb2), and elongin C (Tceb1), indicating that ASB2β E3 ligase complexes were indeed pulled down. Other UPS-related proteins were also associated with WT ASB2β, including several proteasome subunits (Psma2, Psma3, Psma6, Psmb1, Psmb4, Psmb5, and Psmb6), five DUBs (Senp7,Usp10, USP19, Usp38, and Usp39) and, several other E3 ligase proteins, including the predominantly cytosolic E3s, Trim54 (MuRF3) and Trim55 (MuRF2), and the nuclear E3, Trim28 (supplemental Table S3). Importantly, Filamin A (Flna), a previously identified target substrate (22, 55, 85, 86, 87, 88), was also associated with WT ASB2β. Combined, these results suggest the possibility of ASB2β E3 ligase complexes being located in both the cytosolic and nuclear compartments. Consistent with this hypothesis, GO term analysis of all proteins pulled down with WT ASB2β revealed significant overrepresentation of proteins from the cytoplasm, including those involved in translation initiation and cell–cell adhesion, and from the nucleus, including proteins involved in mRNA processing and RNA splicing (Fig. 8*D*). Furthermore, when cell compartment GO analysis was performed only on the top 100 proteins with the highest Log_2_ enrichment, all significantly overrepresented GO terms related to the nucleus (supplemental Fig. S5*A*). These data were supported by the KEGG pathway analysis of all enriched proteins, which identified the terms spliceosome, RNA transport, DNA replication, ribosome, and proteasome as being overrepresented (Fig. 8*D*).

Immunoprecipitation and analysis of all the proteins from cells expressing the Flag-tagged dSOCS ASB2β mutant determined that 535 proteins were significantly enriched compared with cells administered the CON plasmid (q < 0.05 and Log_2_ < −0.5 & >±0.5; Fig. 8*C*), with 84 of those proteins not being enriched with WT ASB2β (supplemental Table S3) (Of note, Cul5, Rbx2 (Rnf7), elongin B (Tceb2), and elongin C (Tceb1) were not significantly enriched with dSOCS ASB2β; Fig. 8*C*). Therefore, the large majority (∼84%) of the proteins associated with ASB2β *per se* were also associated with the larger ASB2β/Cul5/ElonginB-C/Rbx2 complex. Of the 451 proteins common to both WT ASB2β and dSOCS ASB2β, many were related to the biological processes of RNA splicing, mRNA processing, and DNA replication (Fig. 8*E*). Indeed, cell compartment GO analysis of the top 100 enriched proteins, based on Log_2_ fold change, identified eight out of ten terms related to the nucleus (supplemental Fig. S5*B*). In contrast, most of the proteins associated with translation initiation/translation were lost from pull-down of dSOCS ASB2β compared with WT ASB2β (Fig. 8*E*); however, interestingly, several ribosomal proteins (Rpl9, Rpl22, Rpl22l1, Rpl23, Rpl38, Rps7, Rps14, Rps27, Rps27a, Rps27 l) remained associated with the dSOCS ASB2β mutant, as did the nuclear E3 ligase, Trim28. Flna, which was enriched with WT ASB2β, was also significantly enriched (*p* < 0.05) with dSOCS ASB2β; however, it failed to reach the required 0.5 Log_2_ fold change (Log_2_ FC = 0.26; supplemental Table S3). Combined, these data suggest that ASB2β complexes are likely located in both the cytosol and the nucleus and that ASB2β, independent of Cul5/ElonginB-C/Rbx2, is associated with numerous proteins involved in several processes, including translation and mRNA processing, splicing, and transport.

If ASB2β is, in part, localized to the nucleus, then it would presumably contain some type of nuclear localization sequence (NLS). While Cul5 is well known to have an NLS and be localized to the nucleus (89), we were unable to find any published data on an NLS for ASB2β. To investigate this concept further, we used the cNLS Mapper algorithm for calculating predicted NLSs specific for the αβ importin system (http://nls-mapper.iab.keio.ac.jp/cgi-bin/NLS_Mapper_form.cgi) (90). Using the mouse WT ASB2β amino acid sequence (UniProt # Q8K0L0), this analysis of the entire protein identified several putative bipartite NLSs, including a C-terminal sequence, with scores of between 3.0 and 4.0, which is indicative of a protein that is localized to both the nucleus and cytoplasm (supplemental Fig. S5*A*; http://nls-mapper.iab.keio.ac.jp/cgi-bin/NLS_Mapper_help.cgi). Moreover, even with the C-terminal deletion of the SOCS box domain (dSOCS), several putative internal NLSs remained (supplemental Fig. S5*B*). While these data support our novel finding that the ASB2β complex, and ASB2β *per se*, associates with cytoplasmic and nuclear proteins, further mechanistic studies are recommended to definitively confirm the precise ASB2β NLS.

Finally, to identify potential direct ASB2β target substrates, we integrated the proteins from C2C12 cells that were associated with both WT and dSOCS ASB2β with proteins from TA muscles *in vivo* that underwent a significant increase in ubiquitination with ASB2β overexpression (supplemental Table S4). This analysis identified eight ASB2β-associated proteins with increased ubiquitinated peptides (Fig. 8*F*). Of these differentially ubiquitinated candidates, three proteins exhibited an increase in abundance *in vivo* (highlighted in gray in Fig. 8*F*), while the abundance of the remaining five was not altered by ASB2β expression (supplemental Table S4). These proteins include proteins, Signal Transducer and Activator of Transcription 1 (Stat1), N-Myc and STAT interactor (Nmi), lamin B1 (Lmnb1), and Lamin-Associated Protein 1B (Tor1aip1). Other proteins included the largely ER localized TAP-binding protein (Tapbp), the large ribosomal subunit protein 22 (Rpl22), and the GTPase/G-protein-related protein, Guanylate Binding Protein 7 (Gbp7). The one remaining protein identified was Lim-domain only 7 (Lmo7), which binds to the nuclear envelope protein, emerin (91), and to cell adhesion molecules (92). Interestingly, Lmo7, also contains an F-box domain and is, therefore, a putative E3 ligase [also known as Fbxo20; (93)]. Overall, these analyses have identified nuclear and cytosolic proteins that are associated with the ASB2β E3 ligase complex and a subset of nuclear and cytosolic proteins associated with ASB2β independent of Cul5/ElonginB-C/Rbx2. These findings provide a valuable foundation on which to pursue further molecular studies to establish these candidates as substrates for ASB2β E3 ligase activity and to explore the impact of ASB2β-mediated ubiquitination upon protein function.

## Discussion

This is the first study to quantitatively investigate changes to the global skeletal muscle proteome and ubiquitinome in response to increased expression of an E3 ligase. We have shown that the muscle-specific E3 ligase, ASB2β, induces progressive atrophy and an impairment in the intrinsic force producing capability in skeletal muscle, which is associated with changes in the abundance, and ubiquitination, of proteins involved in energy metabolism, muscle contraction, protein synthesis and degradation, and cytoskeletal/sarcomere structure. These data suggest that extensive structural remodeling and altered energy metabolism occur during ASB2β-mediated muscle atrophy, which is influenced at least in part by changes in the ubiquitination status of key proteins. By mapping the ASB2β interactome in cells of the skeletal muscle lineage, we also identified novel putative ASB2β substrates. These data provide a basis for future examination of mechanisms regulated by ASB2β, as regulators of the skeletal muscle phenotype. Overall, our observations reveal a complex molecular response to the increased expression of ASB2β in skeletal muscle, and that there is no simple relationship between the relative changes in ubiquitination and abundance of specific proteins.

Ubiquitination is one of several protein posttranslational modifications that has been shown to have a range of effects, such as regulating protein function, protein–protein interactions, protein localization, and protein abundance by targeting proteins for degradation by the 26S proteasome (75). While our ubiquitinomic analysis identified lysine residues that had a trypsin-generated diglycine tag, which allowed us to determine if a particular lysine residue had been ubiquitinated, it does not allow us to determine whether the diglycine tag represents a monoubiquitination event or a polyubiquitination event, with either linear ubiquitin chains composed of the same-type ubiquitin lysine linkages or branched chains with individual ubiquitin molecules linked to different ubiquitin lysine residues. Thus, our data highlights the potential complexity of the skeletal muscle ubiquitinome and its dynamic regulation in response to external influences, such as the experimental approaches used in this study. More importantly, these data highlight that our understanding of the skeletal muscle ubiquitinome is still incomplete and that further investigation offers opportunities to gain insight into its functional significance and mechanism(s) of regulation and a greater understanding of ubiquitin biology.

To date, despite one study showing that ASB2β is required for myoblast differentiation (16), very little is known regarding the role of ASB2β in mature skeletal muscle. One early study identified ASB2 expression as being regulated in a circadian manner, suggesting that ASB2-mediated ubiquitination of substrate proteins may be required for normal circadian rhythm in skeletal muscle (94). More recently, we showed that ASB2β was markedly downregulated during Follistatin-mediated skeletal muscle hypertrophy, suggesting that a reduction in ASB2β may be required for certain modes of muscle growth (23). Consistent with this hypothesis, ASB2β expression has also recently been shown to be reduced in a mouse model of cardiac hypertrophy (21), while microarray data from a time course study of differentially expressed genes during chronic mechanical overload-induced muscle hypertrophy reported that ASB2β expression was markedly downregulated 1 day after synergist ablation surgery and remained depressed over the following 14 days of muscle growth [https://www.ncbi.nlm.nih.gov/geoprofiles/114037547; (95, 96, 97)]. In addition, we have also shown that ASB2β expression is reduced in other muscle growth models, such of Smad7 overexpression, and combined activin B and myostatin prodomain overexpression (23, 24). While these findings present support for ASB2β as a negative regulator of muscle mass, it remains to be determined why a reduction in ASB2β might be required for muscle growth.

ASB2β is somewhat unique when compared with the classic muscle-specific E3 ligases, atrogin-1 (Fbxo32) and MuRF1 (Trim63), or the recently identified MUSA1 (Fbxo30), in that the overexpression of ASB2β is sufficient to induce significant atrophy in otherwise healthy muscles *in vivo* (9, 15, 23, 98). Indeed, we have shown in these studies that the atrophic response to ASB2β began relatively rapidly, being detectable at 7 days post-rAAV injection, with muscle mass continuing to progressively decline to at least 84 days postinjection. Furthermore, this muscle atrophy was associated with a reduction in specific force production, suggesting an impairment of the excitation contraction-coupling process, force production by the contractile apparatus, and/or the transmission of force from the sarcomere to cytoskeletal, sarcolemmal, or extracellular matrix structures. Interestingly, our proteomics analysis at 10 days postinjection showed that some of the proteins that were downregulated by increased ASB2β expression belong to biological processes and pathways related to muscle contraction and the myosin complex (Fig. 4*A*). This reduction of proteins involved in sarcomere structure, excitation–contraction coupling, and the myosin complex, particularly the loss of contractile proteins, MHC2d/x and MLC1 (Fig. 4*I*), could explain, in part, the ASB2β-induced reduction in specific force production.

In addition to the reduction of key contraction-related proteins, many proteins that were downregulated by ASB2β were related to processes and pathways relevant to mitochondria and mitochondrial function (Fig. 4*A*), including proteins involved in fatty acid oxidation, the TCA/Krebs cycle, and the electron transport chain (ETC; Fig. 4*B*). Interestingly, our western blot analysis found that the decline in mitochondrial proteins was likely a dynamic event, with the decrease occurring between 7 and 10 days and returning to normal by 84 days (Fig. 4, *C*–*F*). We also found that the decrease in mitochondrial protein was preceded by an increase in AMPK T172 phosphorylation at 7 days (Fig. 4*G*), suggesting the potential for an early ASB2β-induced disturbance in the bioenergetic status of the muscle that could have played a role in the reduction of mitochondrial protein content that was first detected in the 10-day proteomics analysis. Interestingly, the gradual return of mitochondrial protein levels to normal by 84 days was associated with a decrease in AMPK T172 phosphorylation. Although we do not yet understand the mechanism that triggered the early decrease in mitochondrial proteins, recent studies have shown that mitochondrial dysfunction, AMPK activation, and changes to cytoskeleton stability can trigger an imbalance in mitochondrial dynamics by inducing mitochondrial fission (64, 65, 66, 67, 68, 69, 70). Of relevance to the effects of ASB2β upon muscle mass, sustained mitochondrial fission can play a role in promoting skeletal muscle atrophy [for recent review see (62)]. Thus, our findings that ASB2β induced an early activation of AMPK, a reduction in mitochondrial fusion-promoting Opa1 protein, an accumulation of cytoskeletal proteins and other proteins involved in regulating cytoskeletal dynamics, and increased the S616 phosphorylation of the fission mediator, Drp1 (Fig. 4*H*), provide compelling evidence that increased ASB2β expression may induce an imbalance in mitochondrial dynamics that could play a role in ASB2β-induced muscle atrophy. These findings provide a basis on which to develop further studies to elucidate the exact mechanism(s) involved.

A decrease in mitochondrial protein abundance may be due to a reduction in mitochondrial biogenesis and/or an increase in mitochondrial degradation, possibly *via* autophagy/mitophagy. While our proteomics analysis did not detect changes to proteins directly involved in mitophagy (*e.g.*, Pink1 and the E3 ligase, Parkin), the two E2 conjugating enzymes that were upregulated by ASB2β (Ube2d3 and Ube2n; Fig. 3*B*) have been reported to play a role in Parkin-dependent mitophagy, with Ube2n mediating K63 ubiquitination of mitochondrial proteins (99). The autophagy receptor, optineurin (Optn), which plays a role in parkin-mediated mitophagy (100, 101), was also upregulated by ASB2β (supplemental Table S1). Furthermore, the proteomics analysis detected the early upregulation of several proteins involved in autophagy (*i.e.*, Ankrd13a, Bag3, Hspa8, Sqstm1/p62, Ubxn4, Ufd1l, USP13, and Vcp), while our Western blot analysis showed an increased expression of the class III PI3 kinase, Vps34 (Fig. 3*D*), and the LC3II/I ratio (Fig. 3*E*), further supporting the possibility of increased autophagy/mitophagy mediated by ASB2β expression.

A significant proportion of downregulated mitochondrial proteins also underwent a relative increase in ubiquitination on specific lysine residues (Fig. 6*D*), which could suggest that their decreased abundance may be due to proteasome-mediated degradation, although as mentioned above, we cannot determine whether these ubiquitination events were the type that typically leads to UPS-mediated degradation (*e.g.*, linear K48-link ubiquitin chains). Regardless of the type of ubiquitination events, it is interesting to consider the questions of when and how did the mitochondrial proteins become ubiquitinated, and what is their fate? Obviously, peripheral outer mitochondrial membrane (OMM)-associated proteins, or trans-OMM proteins with cytoplasmic domains, can be accessed by cytoplasmic E3 ligases (and DUBs); however, what about the intramitochondrial proteins in the intermembrane space, the inner membrane, or the matrix? While mitochondria do not contain proteasomes (102), there is evidence that, in addition to cytosolic OMM-associated E3 ligases (103, 104), they also contain intramitochondrial E3 ligases (105). Therefore, it is possible that some of the identified proteins may have been oxidatively damaged or improperly folded and subsequently ubiquitinated inside the mitochondria, followed by retro-translocation out of the mitochondria for proteasome-mediated degradation (106). On the other hand, nuclear encoded proteins may have been ubiquitinated during translation, or as a precursor protein prior to, or during, OMM translocation. Interestingly, recent evidence has shown that the ubiquitination status of mitochondrial precursor proteins may regulate their TOM complex-mediated importation across the OMM, with increased ubiquitination redirecting some proteins to the proteasome (103, 104). Clearly, further work is required to determine: 1) what are the specific types of ubiquitination events on these mitochondrial proteins; 2) what effect these ubiquitination events have on mitochondrial protein function and fate; and 3) whether or not ASB2β is directly responsible for the changes in mitochondrial protein ubiquitination.

In relation to the last of these questions, an important finding in this study is that the muscle atrophy and dysfunction associated with increased ASB2β expression and the changes in protein ubiquitination are unlikely to be solely due to ASB2β *per se*. Indeed, ASB2β induced an increase in the expression of several other E3 ligases, including Fbxo22, Fbxo30 (MUSA1), Fbxo40, Hectd1, Hectd3, Klhl30, Klhl31, Klhl40, Klhl41, March7, MuRF2 (Trim55), Ube4a, and Ubr3, and a reduction in ASB14 and Ube3c (although none of these are known, or predicted, to be mitochondrially targeted, http://mitominer.mrc-mbu.cam.ac.uk/release-4.0/begin.do) (Figs. 3*B* and 4I). Furthermore, we identified several other nonmitochondrial targeted E3 ligases and DUBs, as having altered levels of ubiquitination, which has the potential to also differentially regulate their activity [Fig. 5; for reviews see (107, 108, 109)]. Of the differentially regulated E3 ligases, very little is known about the roles of ASB14, Fbxo22, Hectd1, Hectd3, Klhl30, March7, Ube3c, Ube4a, or Ubr3 in muscle tissue; however, Klhl31 has been shown to be a negative regulator of MAPK-mediated signaling (110), Fbxo40 is known to target IRS-1 for degradation and potentially inhibit downstream PI3K/AKT/mTORC1 signaling (111) [which is consistent with our finding of suppressed mTORC1 signaling (Fig. 3*C*)], while MUSA1 (Fbxo30) is known to be upregulated during denervation-induced muscle atrophy (15). Thus, ASB2β-induced muscle atrophy and dysfunction are likely the result of a combination of actions of ASB2β *per se*, and other molecular mechanisms regulated, in part, by other E3 ligases and their downstream targets. Importantly, this concept may help to explain why ASB2β overexpression is sufficient to induce muscle atrophy in healthy muscles.

Of the three-remaining upregulated E3 ligases, Klhl40, Klhl41, and MuRF2 (Trim55), all are known to play roles in cytoskeletal and sarcomeric structure. Specifically, Klhl40 regulates myogenesis and binds to and stabilizes nebulin (112, 113), Klhl41, which itself is positively regulated by polyubquitination, plays an important role in maintaining sarcomere integrity by stabilizing structural proteins, such as nebulin, and preventing aggregate formation (46), while MuRF2 is required for maintaining the structure of microtubules, the intermediate filament proteins, desmin and vimentin, and the sarcomeric M-line (114, 115). In this context, it is interesting to note that ASB2β overexpression also induced an increase in several proteins involved in sarcomere organization, the Z disc, microtubules, and the cytoskeleton, including desmin (Des), filamin C (Flnc), nebulin-related anchoring protein (Nrap), obscurin (Obscn), telethonin (Tcap), ankyrin repeat domain-containing protein 2 (Ankrd2), cysteine and glycine-rich protein 3 (Csrp3; aka cardiac LIM protein), myopalladin (Mypn) and xin actin-binding repeat-containing proteins 1 and 2 (Xirp1 and Xirp2) (Fig. 3*F*), while there was a decrease in myosin-binding protein C1 (Mybpc1) (Fig. 4*D*). These data suggest that ASB2β-induced muscle atrophy was associated with marked remodeling of the cytoskeleton and sarcomeres, a phenomenon that is consistent with other models of muscle atrophy, such as muscle denervation (116), and suggest that E3 ligases, including ASB2β, Klhl40, Klh41, and MuRF2, may play roles in this mode of adaptation.

Given this hypothesis of a role for E3 ligases in ASB2β-induced cytoskeletal/sarcomeric remodeling, it is reasonable to expect changes to the ubiquitination status of cytoskeletal and sarcomeric proteins. Indeed, our ubiquitinomic analysis identified that all of the aforementioned upregulated proteins underwent significant alterations to their ubiquitination status at specific lysine residues, as did other structural proteins that did not undergo a change in their abundance, including titin (Ttn) and nebulin (Neb) (Fig. 7). Interestingly, in parallel to increased lysine ubiquitination, other lysine residues simultaneously underwent a decrease in their ubiquitination status, suggesting a role for increased DUB activity in the ASB2β-induced changes to the ubiquitinome. In this context, it is interesting to note that ASB2β expression induced an increase in the abundance of the DUBS, Atxn3, Otulin, Psmd7, and USP13 (Fig. 3*B*). While some cytoskeletal/sarcomeric proteins experienced relatively minor changes in ubiquitination, others demonstrated more marked changes (Fig. 7). For example, we identified titin (Ttn) as having extensive increases in ubiquitination, particularly in the C-terminal half of the protein that corresponds with the region within the sarcomere where actin and myosin filament overlap (*i.e.*, A-band) and close to where titin interacts with the M-band. Of note, the A-band region is also where the E3 ligases, MuRF1 (Trim63) and MuRF2 (Trim55), interact with titin, suggesting that these E3 ligases may play a role in the titin’s ubiquitination and that of other nearby cytoskeletal and sarcomeric proteins (83). Given that this marked increase in titin ubiquitination was not accompanied by a change in total titin abundance, it remains to be determined whether these ubiquitination events may have altered titin, and/or sarcomere, structural and functional properties and contributed to the ASB2β-induced muscle phenotype.

To date, only a limited number of putative ASB2β target substrates have been identified in muscle tissues, including desmin and filamins A and B (16, 21, 22). Therefore, in an attempt to identify other potential targets, specifically in skeletal muscle cells, we first investigated the ASB2β interactome by immunoprecipitating Flag-tagged WT ASB2β, and a Flag-tagged mutant of ASB2β lacking a SOCS box domain, from C2C12 myotubes, and used label free proteomics to identify enriched interacting proteins compared with the control conditions (Fig. 8*A*). Using this approach, we were able to identify proteins that interact with the larger ASB2β/Cul5/ElonginB-C/Rbx2 complex (Fig. 8, *B* and *D*) and proteins associated with ASB2β, independent of the SOCS box-binding Cul5/ElonginB-C/Rbx2 complex (*i.e.*, dSOCS ASB2β; Fig. 8, *C* and *E*). GO analysis revealed the WT ASB2β complex was associated with a range of cytoplasmic proteins, such as translation initiation factors, and ribosomal, and cell adhesion proteins, and also a range of nuclear proteins involved processes such as RNA processing, splicing, and transport (Fig. 8*D*). Furthermore, as expected, the dSOCS ASB2β mutant was associated with a reduced number of different proteins than the WT complex; however, it was still associated with both cytoplasmic and nuclear proteins, including those in biological processes such as RNA splicing and processing (Fig. 8*E*). The enrichment of ribosomal proteins with dSOCS ASB2β (and WT ASB2β) suggests that ASB2β may be associated with ribosomes in the cytoplasm and/or in the nucleolus, where ribosomes are assembled (117). Furthermore, the essential absence of mitochondrial proteins associated with dSOCS ASB2β suggests that the downregulation of mitochondrial proteins with increased ASB2β expression *in vivo* is not due to ASB2β *per se*, but to other mechanisms secondary to ASB2β. When considered together, these novel interactome data suggest that ASB2β is located in the cytoplasm and nucleus of skeletal muscle cells, a finding that is supported by our initial *in silico* analysis of putative ASB2β NLSs (supplemental Fig. S5).

We hypothesised that not all proteins that were found to be associated with ASB2β, independent of Cul5/ElonginB-C/Rbx2, may be direct target substrates of ASB2β. Therefore, in order to more stringently develop a list of priority candidates, we compared proteins from TA muscles *in vivo* that underwent an increase in ubiquitination with ASB2β expression, to the proteins from C2C12 cells that were enriched with both WT ASB2β and dSOCS ASB2β. This analysis narrowed the list of putative targets down to eight candidates, which, again, were a mixture of cytoplasmic and nuclear proteins (Fig. 8*F*). Of these potential ASB2β targets (Fig. 8*F*), at least three are associated with the nuclear envelope, including nuclear intermediate filament, lamin B1 (Lmnb1), Lamin-Associated Protein 1B (Tor1aip1), and Lim-domain only 7 (Lmo7; aka Fbxo20), which binds to the nuclear envelope protein, emerin, and can inhibit TGFβ and fibrosis in smooth muscle (118). Guanylate Binding Protein 7 (Gbp7), which also increased in abundance in muscles overexpressing ASB2β, is a GTPase that belongs to the interferon (IFN)-induced GTPases of the dynamin superfamily (119). Although little is known about the role of Gbp7 in skeletal muscle, its gene expression has been found to be upregulated in a mouse model of muscle hypertrophy induced by activin/myostatin inhibition (120). Other putative ASB2β target proteins include the transcription factor, Stat1, and the Stat interacting cofactor, N-Myc and Stat Interactor (Nmi), both of which also underwent an ASB2β-induced increase in total abundance *in vivo*. These results suggest that ASB2β may play a role in regulating Jak/Stat signaling, which is interesting in light of observations that ASB2 ubiquitinates and targets Jak2 for degradation in nonmuscle cells (121).

Finally, consistent with previous studies of ASB2 targets in different cell types (16, 21, 22, 85, 86, 87, 88), we found that WT ASB2β was associated with filamin A (Flna); however, this association did not quite reach significance with dSOCS ASB2β (supplemental Table S3). This finding suggests that the binding of Flna to ASB2β *per se* may require components of the larger ASB2β complex (*e.g.*, Cul5, Rbx2, Eloc, or Elob). Nevertheless, it is interesting to note that the abundance of both Flna (and Flnc) was increased with ASB2β overexpression *in vivo* (Fig. 3, *G* and *H*, respectively). These observations may mean that ASB2β-induced ubiquitination of Flna: 1) does not lead to its UPS-mediated degradation, 2) leads to Flna ubiquitination but UPS-mediated degradation is somehow impaired leading to an accumulation of these proteins, or 3) that the transcription/translation of Flna was simultaneously upregulated in this atrophic model, such that there was a net increase in these proteins, despite its ASB2β/UPS-mediated degradation. Overall, our novel ASB2β interactome data has significantly expanded the number potential of ASB2β target substrates in skeletal muscle. The findings provide the rationale for future studies to determine which of these candidates are *bona fide* ASB2β targets or targets of other E3 ligases associated with, or upregulated by, ASB2β, and how ASB2β-mediated ubiquitination may affect function and abundance of these proteins.

## Conclusion

In summary, this is the first study to quantitatively investigate changes to the global skeletal muscle proteome and ubiquitinome in response to increased expression of an E3 ligase. Upregulated ASB2β was sufficient to induce progressive skeletal muscle atrophy and reduce intrinsic force-producing capacity, while ASB2β knock-down induced a small, but significant, hypertrophic effect. ASB2β-induced muscle atrophy and contractile dysfunction were associated with the early downregulation of mitochondrial and contractile proteins and the early upregulation of proteins involved in UPS-mediated protein degradation, protein synthesis, and the cytoskeleton/sarcomere. The overexpression of ASB2β results in marked changes in protein ubiquitination (increase and decrease) that are likely a product of both direct ASB2β effects and the influence of other processes initiated as a consequence of the initial effects of ASB2β. Furthermore, we demonstrated that there is not a simple relationship between changes in ubiquitination status and protein abundance. Our studies have revealed that ASB2β appears to interact with cytoskeletal and nuclear proteins, thereby expanding the list of potential ASB2β target substrates that merit further investigation. We propose that it would be informative to examine whether the number of ASB2β targets might be altered in physiological and pathological settings of skeletal muscle adaptation and remodeling. By providing new insight into the complexity of proteome and ubiquitinome changes that occur during E3 ligase-mediated skeletal muscle adaptation, these findings will hopefully support future discoveries into skeletal muscle and ubiquitin biology relevant to health and disease. Our studies have identified novel potential mechanisms by which ASB2 may negatively regulate muscle mass and function. As elevated ASB2 has been associated with the pathological phenotype of type 1 myotonic dystrophy (29), the findings reported herein may offer insight into pathological processes of relevance to this condition.

## Data Availability

The mass spectrometry proteomics data have been deposited to the ProteomeXchange Consortium *via* the PRIDE partner repository with the data set identifiers PXD020040 and PXD023391.

## Supplemental data

This article contains supplemental data (90).

## Conflict of interest

Authors declare no competing interests.
