# Supplementary material for: Dynamic Changes to the Skeletal Muscle Proteome and Ubiquitinome Induced by the E3 Ligase, ASB2β
Source: Mol Cell Proteomics. 2021 Jan 29;20:100050. doi: 10.1016/j.mcpro.2021.100050 (PMC8042406; doi:10.1016/j.mcpro.2021.100050)
Supplement: Supplemental Figures S1–S6 [file mmc1.pdf]

Supplemental Figure 1

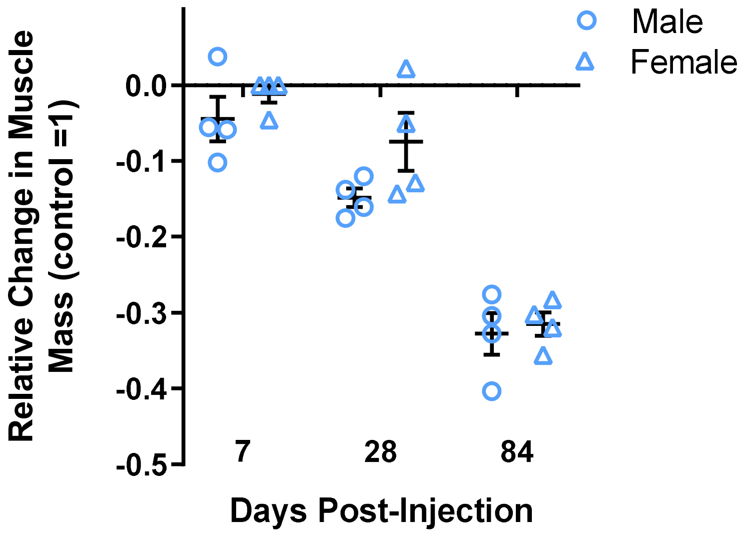

Supplemental Figure 1 – No sex difference in ASB2β-induced muscle atrophy. rAAV vectors encoding Flag-tagged ASB2β or a non-coding control (CON) were injected into the anterior compartment of the lower leg of 8-10 wk male and female C57Bl/6 mice. Tibialis anterior (TA) muscles were dissected at 7, 28 or 84 d post-injection. The ASB2β-induced change in TA muscle mass (MM) is expressed as a ratio with the CON-injected muscles (n = 4/group).

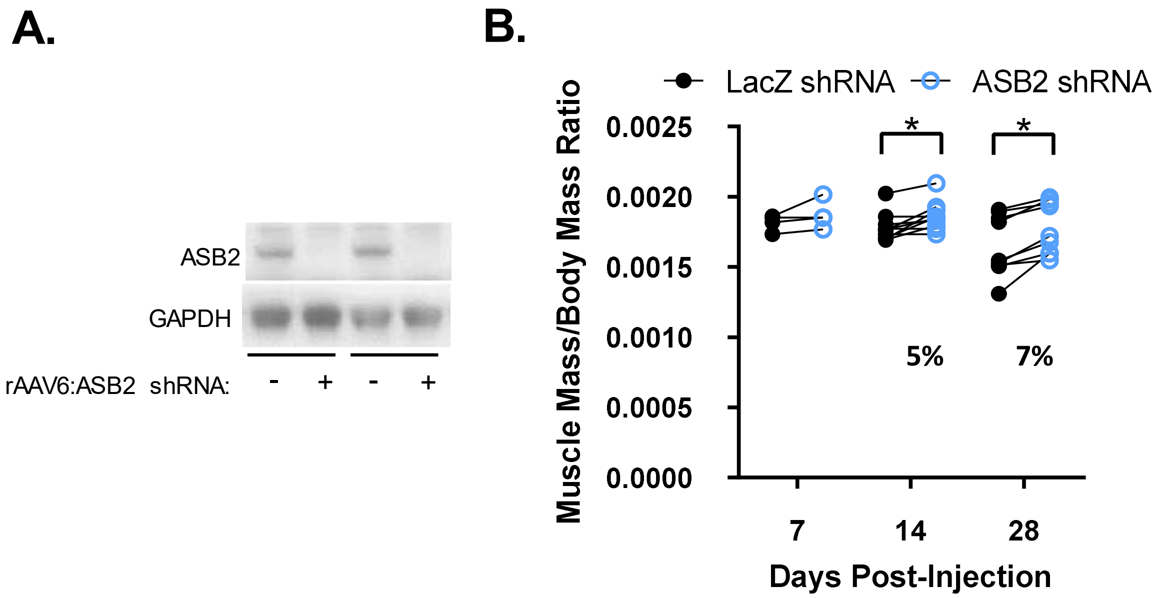

Supplemental Figure 2 – Effect of shRNA -mediated knockdown of ASB2β expression on muscle mass. Two ASB2β shRNAs were individually synthesized within the sequence encoding miR-155 and subcloned in-series into a pAAV6-CMV-humanized Renilla GFP-SV40pA plasmid. rAAV:ASB2β shRNA, or control rAAV:LacZ shRNA, vectors were directly injected into the tibialis anterior (TA) muscles of 8-10 wk male C57Bl/6 mice. TA muscles were dissected at 7, 28 or 84 d post-injection. A) Western blot showing the ASB2β shRNA-induced decrease in ASB2β protein expression. B) The ASB2β shRNA-induced change in TA muscle mass to body mass ratio (n = 4-10/group).

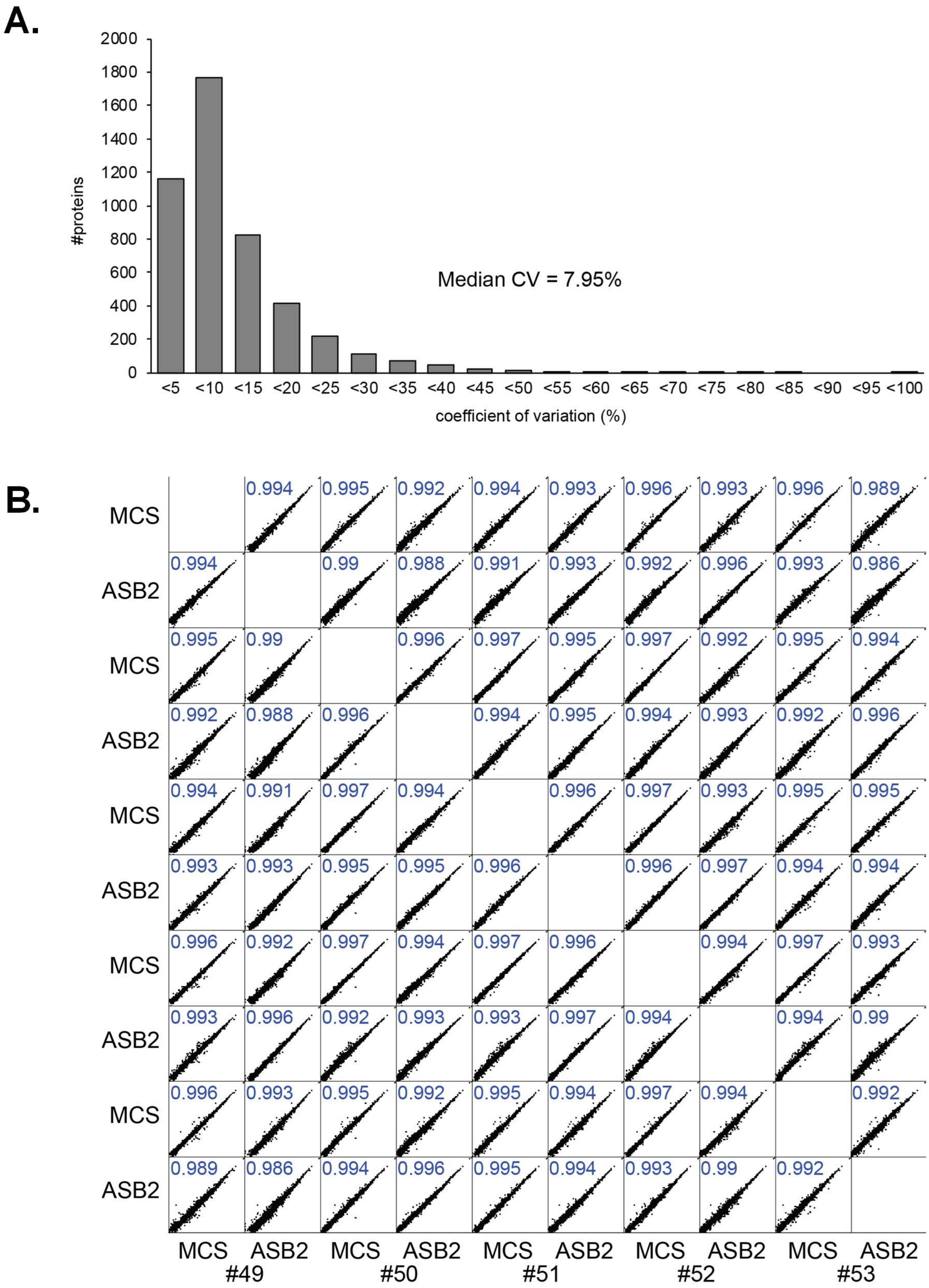

Supplemental Figure 3. (**A**) Histogram highlighting coefficient of variation (CV) of total proteomic data (ratio of ASB2 / MCS) (n=5). (**B**) Scatter-plots highlighting Pearson correlation of the total proteomics data (n=5). Numbers below indicate mouse number. MCS: multiple cloning site negative control.

Supplemental Figure 4

Translation Component	Protein	Log_2 FC	Translation Component	Protein	Log_2 FC
**Initiation Factors**	Eif2a	0.061	**Elongation Factors**	Eef1a1	0.233
	Eif2b3	0.036		Eef2	0.033
	Eif3a	0.029			
	Eif3b	0.113	**60S Ribosome**	Rpl3	0.234
	Eif3e	0.039		Rpl4	0.116
	Eif3i	0.083		Rpl5	0.109
	Eif4a1	0.069		Rpl9	0.127
	Eif4g1	0.182		Rpl12	0.072
	Eif4g3	0.111		Rpl19	0.119
	Eif5	0.087		Rpl27a	0.138
**tRNA-synthetases**	Dars	0.096		Rpl32	0.124
	Eprs	0.133		Rpl36	0.094
	Farsa	0.059		Rpl37a	0.183
	Hars	0.049		Rpl39	0.087
	Iars	0.082			
	Kars	0.116	**40S Ribosome**	Rps11	0.137
	Nars	0.090		Rps26	0.120
	Rars	0.067		Rps27a	0.155
	Vars	0.063		Rpsa	0.115

Supplemental Figure 4 – Specific translation/protein synthesis and ribosomal proteins [and their Log_2 fold change (Log_2FC)], identified as significantly enriched by Database for Annotation, Visualization and Integrated Discovery (DAVID)-based pathway enrichment gene ontology (GO) analysis of proteins upregulated by 10 d of ASB2β overexpression (see Suppl. Table 1).

Supplemental Figure 5

A.

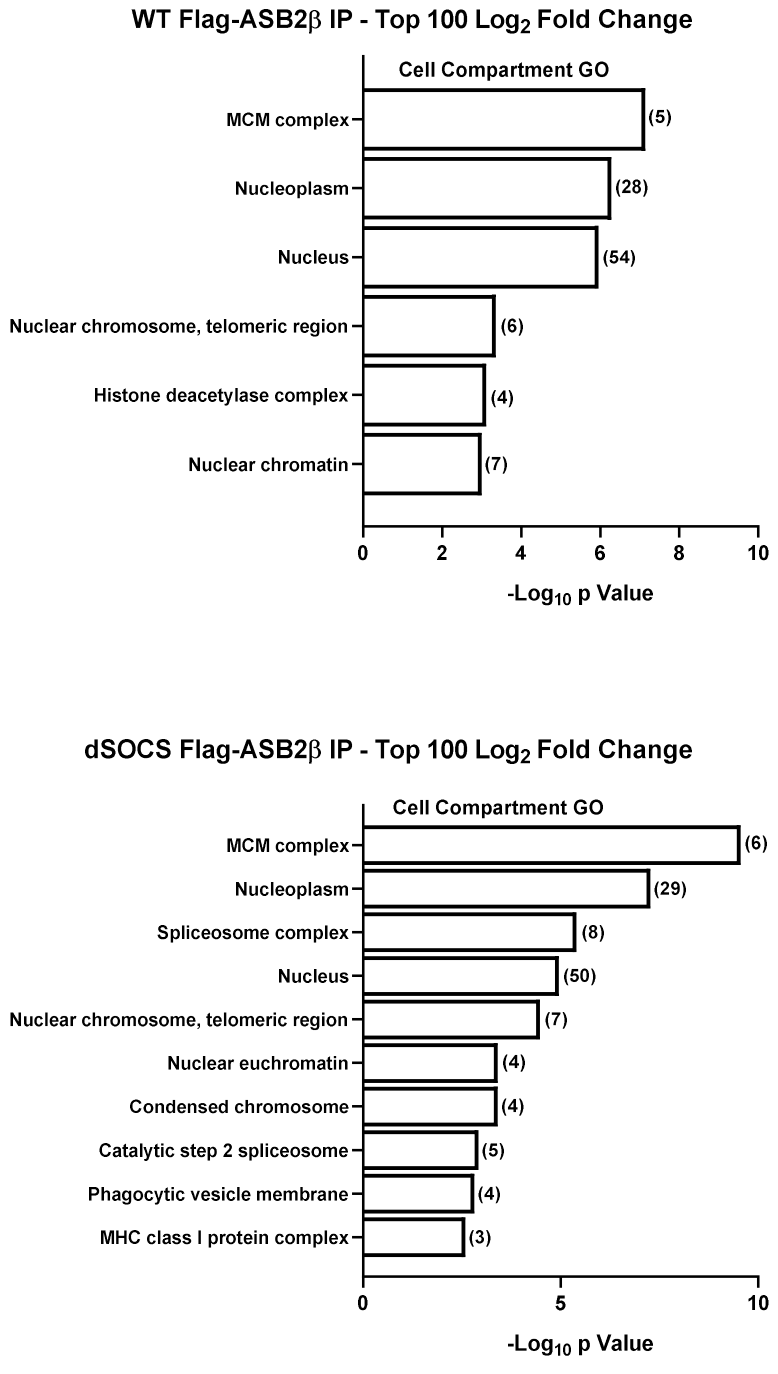

Supplemental Figure 5 – Cell Compartment Gene Ontology (GO) term analysis of the top 100 most highly enriched proteins with Flag-tagged WT and dSOCS ASB2β immunoprecipitation (IP) from C2C12 myotubes. A & B) Database for Annotation, Visualization and Integrated Discovery (DAVID)-based cell compartment enrichment gene ontology (GO) term analysis of proteins enrichment with WT ASB2β IP **(A)** and dSOCS ASB2β **(B).** GO terms are plotted against the $-\text{Log}_{10}$ *P* value (in brackets are the number of proteins included in each GO term). The top 100 enriched proteins for WT and dSOCS ASB2β was based on the highest Log_2 fold change (see Suppl. Table 3)

Supplemental Figure 6

A. **WT ASB2β**

cNLS Mapper Result

Predicted NLSs in query sequence

```
MATEISTRGRQRAIGHEEYSLYSSLSEEELLQMAIEQSLADKTRGPTPAE 50
ASASSQTNHQPGHFHPWTRSPSSPENPPARAPLGLFQGVMQKYSSNLFKT 100
SQMAAMDPVLKAIKEGDEEALKIMIQDGKNLAEPNKEGWLPLHEAAYYGQ 150
LGCLKVLQQAYPGTIDQRTLQEETALYLATCREHLDCLLSLLQAGAEPDI 200
SNKSRETPLYKACERKNAEAVRILVRYNADANHRCNRGWTALHESVSRND 250
LEVMEILVSGGAKVEAKNVYSITPLFVAAQSGQLEALRFLAKHGADINTQ 300
ASDSASALYEASKNEHEDVVEFLLSQGADANKANKDGLLPLHVASKKGNY 350
RIVQMLLPVTSRTRVRRSGISPLHLAAERNHDAVLEALLAARFDVNAPLA 400
PERARLYEDRRSSALYFAVVNNNVYATELLLLAGADPNRDVISPLLVAIR 450
HGCLRTMQLLLDHGANIDAYIATHPTAFPATIMFAMKCLSLLKFLMDLGC 500
DGEPCFSCLYGNGPHPPAPRPGRFHDAPVDDKAPSVVQFCEFLSAPEVSR 550
WAGPIIDVLLDYVGNVQLCSRLKEHIDSFEDWAVIKEKAEPPRPLAHLCR 600
LRVRKAIGKYRIKLLDTLPLPGRLIRYLKYENTQ                 634
```

Predicted monopartite NLS

Pos.	Sequence	Score

Predicted bipartite NLS

Pos.	Sequence	Score
133	EPNKEGWLPLHEAAYYGQLGCLKVLQQAYP	3
152	GCLKVLQQAYPGTIDQRTLQEETALYLATC	3
310	EASKNEHEDVVEFLLSQGADANKANKDGLLP	3.4
362	RTRVRRSGISPLHLAAERNHDAVLEALLAAR	3
571	RLKEHIDSFEDWAVIKEKAEPPRPLAHL	3.9
571	RLKEHIDSFEDWAVIKEKAEPPRPLAHLC	3.6
581	DWAVIKEKAEPPRPLAHLCRLRVRKAIGKYRIK	3.2
600	RLRVRKAIGKYRIKLLDTLPLPGRLIRYLKYE	3.2

B. **dSOCS ASB2β**

cNLS Mapper Result

Predicted NLSs in query sequence

```
MATEISTRGRQRAIGHEEYSLYSSLSEEELLQMAIEQSLADKTRGPTPAE 50
ASASSQTNHQPGHFHPWTRSPSSPENPPARAPLGLFQGVMQKYSSNLFKT 100
SQMAAMDPVLKAIKEGDEEALKIMIQDGKNLAEPNKEGWLPLHEAAYYGQ 150
LGCLKVLQQAYPGTIDQRTLQEETALYLATCREHLDCLLSLLQAGAEPDI 200
SNKSRETPLYKACERKNAEAVRILVRYNADANHRCNRGWTALHESVSRND 250
LEVMEILVSGGAKVEAKNVYSITPLFVAAQSGQLEALRFLAKHGADINTQ 300
ASDSASALYEASKNEHEDVVEFLLSQGADANKANKDGLLPLHVASKKGNY 350
RIVQMLLPVTSRTRVRRSGISPLHLAAERNHDAVLEALLAARFDVNAPLA 400
PERARLYEDRRSSALYFAVVNNNVYATELLLLAGADPNRDVISPLLVAIR 450
HGCLRTMQLLLDHGANIDAYIATHPTAFPATIMFAMKCLSLLKFLMDLGC 500
DGEPCFSCLYGNGPHPPAPRPGRFHDAPVDDKAPSVVQFCEFLSAPEVSR 550
WAGPIIDVLLDYVGNVQLCSRLKEHIDSFEDWAV                 584
```

Predicted monopartite NLS

Pos.	Sequence	Score

Predicted bipartite NLS

Pos.	Sequence	Score
133	EPNKEGWLPLHEAAYYGQLGCLKVLQQAYP	3
152	GCLKVLQQAYPGTIDQRTLQEETALYLATC	3
310	EASKNEHEDVVEFLLSQGADANKANKDGLLP	3.4
362	RTRVRRSGISPLHLAAERNHDAVLEALLAAR	3

cNLSs are conventionally defined as having one (monopartite) or two clusters of basic amino acids separated by a 9-12 amino acid linker (bipartite)

cNLS Mapper

http://nls-mapper.iab.keio.ac.jp/cgi-bin/NLS_Mapper_form.cgi

Kosugi et al. (2009), Systematic identification of cell cycle-dependent yeast nucleocytoplasmic shuttling proteins by prediction of composite motifs. PNAS. 106:10171-10176.

Supplemental Figure 6 – Identification of putative nuclear localization sequences in WT and dSOCS ASB2β proteins. cNLS Mapper (http://nls-mapper.iab.keio.ac.jp/cgi-bin/NLS_Mapper_form.cgi) was used to predict nuclear localisation sequences (NLS) in wild type (WT) (A) and SOCS-box deleted (dSOCS) (B) ASB2β proteins. The cNLS Mapper Results boxes depict the amino acid sequence of the WT and dSOCS proteins with the putative NLSs highlighted in red font. The Predicted bipartite NLS boxes show the predicted bipartite NLS sequences and their respective score which indicates the strength of the NLS. A score of 3-4 predicts that the protein is localized to both the cytoplasm and nucleus (http://nls-mapper.iab.keio.ac.jp/cgi-bin/NLS_Mapper_help.cgi).
